# Stability of biomaterials used in adjunct to coronally advanced flap: A systematic review and network meta‐analysis

**DOI:** 10.1002/cre2.461

**Published:** 2021-11-29

**Authors:** Sourav Panda, Shahnawaz Khijmatgar, Heber Arbildo‐Vega, Abhaya Chandra Das, Manoj Kumar, Mohit Das, Leonardo Mancini, Massimo Del Fabbro

**Affiliations:** ^1^ Department of Periodontics and Oral Implantology Institute of Dental Sciences, Siksha O Anusandhan (Deemed to be) University Bhubaneswar Odisha India; ^2^ Department of Biomedical, Surgical and Dental Sciences University of Milan Milan Italy; ^3^ Department of Oral Biology and Genomic Studies Nitte (Deemed to be University), AB Shetty Memorial Institute of Dental Sciences Mangalore India; ^4^ Department of General Dentistry, Dentistry School Universidad San Martín de Porres Chiclayo Peru; ^5^ Department of life health and environmental sciences University of L'Aquila Italy; ^6^ IRCCS Istituto Ortopedico Galeazzi Milan Italy

**Keywords:** CAF, clinical attachment level, keratinised tissue width, network meta‐analysis, oral, recession height, recession width, regeneration, root coverage, soft tissue, systematic review

## Abstract

**Aim:**

The objective of this network meta‐analysis was to rank different biomaterials used in adjunct to coronally advanced flap (CAF), based on their performance in root‐coverage for Miller's Class I and II gingival recessions.

**Materials and methods:**

An electronic database search was carried out in PUBMED, CENTRAL, SCOPUS, and EMBASE to identify the eligible articles and compiled into the citation manager to remove the duplicates. The primary outcome was keratinized gingival tissue width (KGW) and percentage of root coverage (%RC). The treatment effect of different biomaterials was estimated using predictive interval plots and ranked based on biomaterials performance, using multidimensional scale ranking.

**Results:**

CAF + connective tissue graft (CTG), CAF + platelet concentrate matrix (PCM) and acellular dermal matrix (ADM) ranked at the top positions in performance in improving KGW. The highest ranked materials in improving percentage of root coverage in gingival recession were CAF + collagen matrix (CM) + gingival fibroblasts (GF), CAF + ADM + platelet rich plasma (PRP) and CAF + ADM, as compared to CAF alone.

**Conclusion:**

CTG, ADM, platelet concentrates, and CM + GFs, when used in adjunct to CAF, showed improved stability over ≥12 months of follow‐up, better percentage of root coverage, and improved keratinized gingival width.

## 1 INTRODUCTION

Gingival recession (GR) is characterized by displacement of the gingival margin below the level of cemento‐enamel junction (Cortellini & Bissada, 2018). Several etiological factors like age, anatomical, physiological, pathological, trauma, hygiene, abnormal frenal attachment, and so on, were identified for this condition (Fu et al., 2012) which may account for its relatively high incidence in the population (Rios et al., 2014; Susin et al., 2004). It affects more than 50% of population including healthy individuals. Recession of 1 mm or more is prevalent in aged 30 years and older. The risk increases with age. Root exposure leads to hypersensitivity, cervical caries, aesthetics complications, and non‐carious cervical lesions (Jepsen et al., 2017).

Root coverage procedures (RCP) have shown to be effective in treating single and multiple GRs (Cairo et al., 2014; Tavelli, Barootchi, et al., 2018; Tavelli, Barootchi, et al., 2019; Tavelli, Ravidà, et al., 2019), and in literature, several techniques were proposed. Nevertheless, the superiority of coronally advanced flap (CAF) combined with connective tissue graft (CTG) is clear. Indeed, CAF + CTG is a gold standard in RCP for the best outcomes achieved in terms of mean root coverage, keratinized tissue width, gingival thickness and aesthetics results (Zucchelli et al., 2020).

However, the presence of a second surgical site, patient morbidity, and limited availability are the main drawbacks that have been largely described for CTG (Tavelli, Asa'ad, et al., 2018; Tavelli, Barootchi, et al., 2019; Tavelli, Ravidà, et al., 2019). For this reason, several CTG substitutes were introduced including Platelet rich plasma (PRP) or Platelet rich fibrin (PRF), acellular dermal matrix (ADM), enamel matrix derivate (EMD), and xenogeneic collagen matrix (CMX). These biomaterials suffer from limitations in term of shape, consistency and size. PRF is a living cellular graft enriched with growth factors and it is a good alternative to CTG for the availability and the easy handling (Dohan et al., 2006; Miron et al., 2020). The ADM is soft CTG generated by a de‐cellularization process preserving the extracellular skin matrix with high costs and it is not in use for ethical problems in diverse countries (McGuire et al., 2020; Tavelli et al., 2020). EMD is a porcine fetal tooth material extracted and manipulated as a gel used as an enhancer in oral regenerative procedures (McGuire & Nunn, 2003). CMX is another biomaterial, which has different layers of collagen fibers and porous layer facilitating blood clot formation and in‐growth of tissue from adjacent sites (McGuire & Scheyer, 2016; Vignoletti et al., 2011).

Several studies revealed the comparison among CAF + CTG and each alternative therapy during a follow‐up period of 6–12 months showing divergent results (Keceli et al., 2015; McGuire & Scheyer, 2010; Tonetti et al., 2018). According to the authors in the literature, a direct, indirect and mixed evidence for all these biomaterials contributing to the success of root coverage is not present and data extracted from non‐systematic comparisons might be confusing and not well interpreted. In addition, the data from all studies are heterogeneous (differences in estimates of effect across studies that assessed the same comparison), which makes difficult to compare all materials. A conventional pairwise meta‐analysis results in only one pooled effect estimate. Therefore, a novel method of weighing the effect estimate through network meta‐analysis (NMA) has been proposed.

Previous network systematic reviews tried to collect data evaluating the clinical advantages for each CTG substitute with several limitations such as the follow‐up period of 6‐months which might be a limit, the inclusion of randomized clinical trials (RCTs) with a high risk of bias influencing results and the inclusion of RCTs with smoker patients or RCTs where the absence or presence of smoker patients was not reported (Buti et al., 2013; Moraschini et al., 2020). Thus, the purpose of this systematic review and NMA was to compare the clinical effects among patients who have one or more gingival recession sites and corrected with intervention of CTG substitutes and compared with controls or CAF alone or in combination for regeneration of keratinised gingival width (KGW), clinical attachment level (CAL), recession width (RW), recession height (RH), pocket depth outcomes during a long follow‐up period.

## 2 MATERIAL AND METHODS

This review was performed in accordance with of PRISMA guidelines. The protocol for this review and NMA was registered in PROSPERO with registration ID: CRD42020208010.

The eligibility of study was decided based on PICO format.

Type of Patients: Patients who had one or more than one site of gingival recession was considered for assessment and further review analysis.

Type of Intervention: CAF or/and CTG Substitutes.

Type of Comparator: Compared with Placebo, Control or CAF or/and combination of CAF + biomaterials or CTG substitutes.

Type of outcomes: KGW, CAL, Recession Height, Recession Width, Pocket Depth were the outcomes.

Type of Duration: More than 6 months' follow‐up periods.

### 2.1 Research question

What is the treatment effect of different biomaterials like CTG, EMD, ADM, PRP/PRF, CMX, and combination of these when used in adjunct to CAF for root coverage?

### 2.2 Search strategy

An electronic database search was carried out in PUBMED, CENTRAL, SCOPUS, and EMBASE to identify the potentially eligible articles using the following strategy:

“((((Coronally advanced flap) OR (CAF)) OR (modified coronally advanced flap)) OR (coronally displaced flap)) AND ((((((((((((Enamel matrix derivative) OR (Connective tissue graft)) OR (Guided tissue regeneration)) OR (Collagen matrix)) OR (Acellular dermal matrix)) OR (platelet rich fibrin)) OR (platelet rich plasma)) OR (PRF)) OR (PRP)) OR (barrier membrane)) OR (amniotic membrane)) OR (hyaluronic acid)) OR (Emdogain)) OR (CTG)).”

A manual search in periodontal journals like Journal of Clinical Periodontology, Journal of Periodontal Research, Journal of Periodontal and Implant Science, International Journal of Periodontics and Restorative Dentistry, and Journal of Periodontology. There were no limits or filters applied during the search. Both studies relevant to the topic in areas of systematic reviews and clinical trials were searched.

### 2.3 Inclusion criteria

1. Randomized Clinical Trials (Both parallel and split mouth design)
2. Study follow up duration at least 12 months
3. Minimum sample size of 10 per group
4. CAF procedure should be employed both in test and control group.
5. The test group should have any of the biomaterials in adjunct to CAF compared to control group with a different biomaterial or none in adjunct to CAF.
6. Treatment of Class 1 and 2 gingival recessions only.
7. Both isolated and multiple recession

### 2.4 Exclusion criteria

1. Studies not in English
2. Study participants under any medication which could influence the outcome of treatment.
3. Teeth with non‐carious cervical lesions (NCCL)
4. Animal studies

### 2.5 Study selection

The studies from the databases searches were compiled into citation manager to remove duplicates and screened for all titles and abstract by two independent reviewers (M.K and A.C.D). The eligible studies were then subjected to full text assessment and included for qualitative assessment. In case of disagreement or uncertainty while selecting the eligible articles, an expert third reviewer (M.D.F) was consulted until a consensus was reached. Detailed reasons were mentioned for all excluded studies.

### 2.6 Data extraction

The qualitative data was extracted using excel spreadsheet. The data extraction was carried out by using two independent reviewers (M.Dd, H.A.V.). In cases like missing or unclear data or need for additional data or raw data, the authors were approached through emails or telephone for enquiring the details of missing or unclear information.

### 2.7 Outcomes

The primary outcomes that were assessed in this review were keratinized gingival width (KGW) and the percentage of root coverage (%RC). The secondary outcomes assessed included CAL, RW, and RD.

### 2.8 Data synthesis

The data extracted were both qualitative and quantitative. The former were related to demographics of the study and type of publication. The quantitative data for the different outcomes allowed to undertake NMA. The NMA enables to develop a network geometry plot, where the number of studies and subjects between the comparators are projected. The risk of bias in each network was also estimated. The predictive interval plots (Prl) were calculated to predict the effects of future clinical studies incorporating heterogeneity. The surface under the cumulative ranking curve (SUCRA) was calculated for each treatment. Treatments were ranked based on their respective performances. Treatments with SUCRA values with higher percentage of being first were ranked higher and values with lower percentage were ranked lower (0–100%). The multidimensional scale ranking was employed to rank the biomaterials based on their dissimilarity. The network estimates for all comparisons are treated as proximity data aimed to reveal their latent structure. By this, the dissimilarity between any two treatments was distinguished. NMA was carried out using Stata version 16 (StataCorp, College Station, TX) by a single reviewer (S.K).

## 3 RESULTS

**Figure 1:**
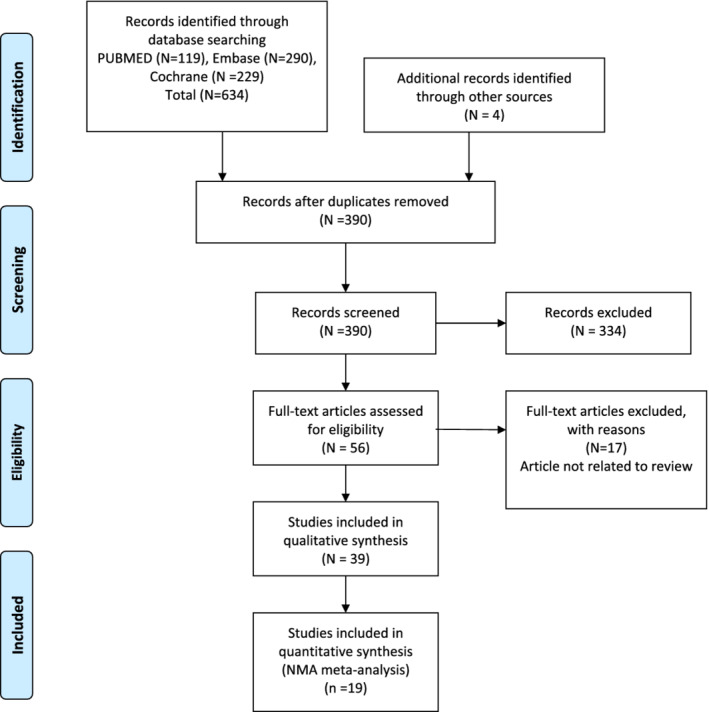
PRISMA flow chart diagram

This qualitative and NMA analysis was carried out by assessing 39 RCTs analyzing the stability of CAF when used alone or in combination with different biomaterials in treatment of class I and II gingival recession defects, over at least 12 months follow‐up. The electronic database search and manual search of related journals and bibliographies yielded a total of 1223 articles. The searches from different databases were imported to a citation manager (ENDNOTE) to identify 938 articles after removing duplicates. All the articles were subjected to title and abstract screening, and were narrowed down to identify 56 potentially eligible studies. These studies were subjected to detailed full text assessment by two independent reviewers. Out of 56 eligible studies, 39 RCTs were included in this systematic review and 19 were considered for NMA. The detailed process of study selection is provided in the PRISMA flow chart (Figure 1). The rest of 17 articles were excluded with detailed reason for exclusion (refer Table 1).

**Table 1:** List of excluded studies

Sl no.	Study	Reason of exclusion
1.	Tavelli, Barootchi, et al. (2019); Tavelli, Ravidà, et al. (2019)	Coronally advanced flap compared to tunnel technique
2.	Stefanini et al. (2018)	Not a randomized clinical trial but a case series
3.	Bellver‐Fernández et al. (2016)	Test group has less than 10 sites
4.	Wang et al. (2015)	Same biomaterial used in both groups
5.	Wang et al. (2014)	Same biomaterial used in both groups
6.	Zucchelli et al. (2014)	Same biomaterial used in both groups
7.	Aroca et al. (2013)	Tunnel technique has been used in both the groups
8.	McGuire et al. (2012)	Less than 10 patients as sample size
9.	Aleksic et al. (2010)	Study in Russian language
10.	Aroca et al. (2009)	Follow up till 6 months
11.	Pourabbas et al. (2009)	Follow up till 6 months
12.	Moses et al. (2006)	Two separate procedures have been compared
13.	Dominiak et al. (2006)	Three separate procedures have been compared
14.	Nemcovsky et al. (2004)	Inclusion of cervically abraded teeth
15.	Wennström et al. (1996)	Non‐randomized, prospective clinical study
16.	Pini‐Prato et al.( 2010)	Non randomized study
17.	Henriques et al. (2010)	Class III gingival recessions

### 3.1 Characteristics of included studies

The demographic and interventional characteristics of all included studies are presented in Tables 2 and 3, respectively.

**Table 2:** Demographic characteristics of included studies

Sl no.	Author and year	Study design	Age in years (range, mean SD)	Type of recession	No. of patients	No. of sites	Test procedure		Control procedure	Follow‐up
1.	Barakat and Dayoub (2020)	RCT split‐mouth	20–45	Single	20	40	CAF + PCM		CAF + CTG	12 months
2.	Rotundo et al. (2021)	RCT parallel	> 18	Multiple	24	61	CAF + CMX		CAF	12 months
3.	Aydinyurt et al. (2019)	RCT split‐mouth	18–55	Single	19	38	CAF + CTG + EMD		CAF + CTG	12 months
4.	Pilloni et al. (2019)	RCT parallel	21–47	Single	30	30	CAF + HA		CAF	18 months
5.	Rotundo et al. (2019)	RCT parallel	> 18	Multiple	24	61	CAF + CMX		CAF	12 months
6.	França‐Grohmann et al. (2019)	RCT parallel	29.74	Single	30	30	CAF + EMD		CAF	12 months
7.	Gürlek et al. (2020)	RCT split‐mouth	> 18	Multiple	15	82	CAF + ADM		CAF + CTG	18 months
8.	Matoh et al. (2019)	RCT split‐mouth	21–52	Single	10	20	CAF + CM		CAF + CTG	12 months
9.	Francetti et al. 2018)	RCT parallel	> 18	Single	20	NR	CAF + CTG		CAF	60 months
10.	Kuka et al. (2018)	RCT parallel	21–41	Multiple	24	52	CAF + PRF		CAF	12 months
11.	Rasperini et al. (2018)	RCT parallel	37–63	Single	25	25	CAF + CTG		CAF	108 months
12.	Çetiner et al. (2018)	RCT split‐mouth	20–67	Multiple	12	84	CAF + ADM + PRP		CAF + ADM	12 months
13.	Abou‐Arraj et al. (2017)	RCT parallel	24–74	Single	17	17	CAF + ADM‐A		CAF + ADM‐B	12 months
14.	Jepsen et al.et al. (2017)	RCT split‐mouth	20–73	Single	18	36	CAF + CMX		CAF	36 months
15.	Cairo et al. (2016)	RCT parallel	20–53	Multiple	32	74	CAF + CTG		CAF	12 months
16.	McGuire & Scheyer, (2016	RCT split‐mouth	51.3 ± 13.9	Single	17	34	CAF + CMX		CAF + CTG	60 months
17.	Godavarthi et al. (2016)	RCT split‐mouth	41.4 ± 7.6	Single	14	28	CAF + PPG		CAF + ADM	12 months
18.	Stefanini et al. (2016)	RCT split‐mouth	39.5 ± 13.8	Multiple	45	41	CAF + CMX		CAF	12 months
19.	Lops et al. (2015)	RCT parallel	46	Single	28	28	CAF + CTG		CAF	12 months
20.	Cairo et al. (2015)	RCT parallel	45.9 ± 10.3	Single	24	24	CAF + CTG		CAF	36 months
21.	Milinkovic et al. (2015)	RCT split‐mouth	18–55	Both	18	36	CAF + AFCC		CAF + CTG	12 months
22.	McGuire et al. (2014)	RCT split‐mouth	29–68	Both	30	60	CAF+ rhPDGF‐BB		CAF + CTG	60 months
23.	Ahmedbeyli et al. (2014)	RCT parallel	22–40	Multiple	24	48	CAF + ADM		CAF	12 months
24.	Zucchelli et al. (2014)	RCT parallel	>18	Multiple	50	50	CAF + CTG		CAF	60 months
25.	Cardaropoli et al. (2014)	RCT parallel	38.4 ± 11.1	Multiple	32	113	mCAF+CM		mCAF	12 months
26.	Alkan and Parlar (2013)	RCT split‐mouth	35–53	Multiple	12	56	CAF + EMD		CAF + CTG	12 months
27.	Kuis et al. (2013)	RCT split‐mouth	20–52	Multiple	37	114	CAF + CTG		CAF	60 months
28.	Köseoğlu et al. (2013)	RCT split‐mouth	19–41	Single	11	22	CAF + CMX + GF		CAF + CMX	12 months
29.	Roman et al. (2013)	RCT split‐mouth	21–28	Both	42	57	CAF + CTG + EMD		CAF + CTG	12 months
30.	Kumar and Murthy (2013)	RCT split‐mouth	18–60	Single	12	24	CAF + PCG		CAF + CTG	12 months
31.	Cordaro et al. (2012)	RCT split‐mouth	18–60	Multiple	10	58	CAF + EMD		CAF	24 months
32.	Cardaropoli et al. 2012)	RCT split‐mouth	21–59	Multiple	18	22	CAF + CMX		CAF + CTG	12 months
33.	Alkan and Parlar (2011)	RCT split‐mouth	23–42	Single	12	24	CAF + EMD		CAF + CTG	12 months
34.	Jankovic et al. (2010)	RCT split‐mouth	21–48	Single	20	40	CAF + EMD		CAF + PRF	12 months
35.	Del Pizzo et al. (2005)	RCT split‐mouth	18–56	Single	15	30	CAF + EMD		CAF	24 months
36.	Spahr et al. (2005)	RCT split‐mouth	23–62	Single	30	60	CAF + EMD		CAF + Placebo	24 months
37.	McGuire and Nunn (2003)	RCT split‐mouth	23–62	Single	20	40	CAF + EMD		CAF + CTG	12 months
38.	Hägewald et al. (2002)	RCT split‐mouth	22–62	Single	36	72	CAF + EMD		CAF + Placebo	12 months
39.	Zucchelli et al. (1998)	RCT parallel	23–33	Single	54	54	CAF + GTR(R) CAF + GTR(NR)		CAF + CTG	12 months

Abbreviations: RCT‐ Randomized controlled trial, CAF‐ Coronally Advanced Flap, mCAF‐ modified Coronally Advanced Flap, PCM‐ Platelet concentrate matrix, CMX‐ Xenogenic Collagen Matrix, CTG‐ Connective Tissue Graft, EMD‐ Enamel Matrix Derivative, HA‐ Hyaluronic Acid, ADM‐ Acellular Dermal Matrix, PRF‐ Platelet Rich Fibrin, PRP‐ Platelet Rich Plasma, ADMA‐ Acellular Dermal Matrix(AlloDerm BioHorizons), ADMB‐ Acellular Dermal Matrix(Puros Dermis Zimmer Biomet), PPG‐ Periosteal Pedicle Graft, AFCC‐ Autologous Fibroblast Cell Culture, rh‐PDGF‐BB – recombinant human Platelet Derived Growth Factor‐ BB, GF‐ Gingival Fibroblasts, PCG‐ Platelet Concentrate Graft, GTR(R)‐ Resorbable Guided Tissue Regeneration Membrane, GTR(NR)‐ None Resorbable Guided Tissue Regeneration Membrane.

**Table 3:** Interventional characteristics of included studies

Author and year	Procedure	Test biomaterial	Type of biomaterial	Trade name ‐ test	Control biomaterial	Type of biomaterial	Trade name ‐ control	Surgery time(test)	Surgery time (control)	Outcomes assessed
Barakat and Dayoub (2020)	CAF	CMX	XENO	Mucograft collagen matrix; Geistlich Pharma	CTG	AUTO	Autologous	NR	NR	RD, PD,CAL, WKG, RC%, CRC%, aesthetic score(RES) and pain intensity
Rotundo et al. (2021)	CAF	CMX	XENO	Geistlich Mucograft®, Geistlich Pharma AG	None	‐	None	NR	NR	PI, FMBS,RD, WKG,GT,PD
Aydinyurt et al. (2019)	CAF	EMD	XENO	Emdogain®, Straumann, Basel, Switzerland	CTG	AUTO	Autologous	NR	NR	RD,RW, RC%, CRC%, aesthetic score (RES)
Pilloni et al. (2019	CAF	HA	ALLP	HyaDENT BG, Bioscience, Germany	None	‐	None	NR	NR	RD, CAL,PD, WKG, CRC%, RC%, pain intensity
Rotundo et al. (2019)	CAF	CMX	XENO	Geistlich Mucograft®, Geistlich Pharma AG	None	‐	None	47.3 ± 5.8	36.1 ± 4.6	RD, RW, BOP, PI, PD, CAL, WKG,GT
França‐Grohmann et al. (2019)	CAF semilunar	EMD	XENO	Emdogain®, Institut Straumann AG, Basel, Switzerland	None	‐	None	NR	NR	RD,RW,WKG, GT, PD, CAL
Gürlek et al. (2020)	Modified CAF	ADM	ALLO	Mucoderm, Botiss Gmbh, Berlin, Germany	CTG	AUTO	Autologous	NR	NR	RD,RW, WKG, PD, CAL
Matoh et al. (2019	CAF	CM	XENO	OsteoBiol Derma, Tecnoss	CTG	AUTO	Autologous	NR	NR	RD, PD, CAL, WKG, GT, RC%
Francetti et al. (2018)	CAF	CTG	AUTO	Autologous	None	‐	None	NR	NR	RD, CRC%, WKG, CAL, RC%
Kuka et al. (2018)	CAF	PRF	BIO	Autologous	None	‐	None	NR	NR	RD, RW, PD, CAL, GT, WKG, PI, GI and BOP
Rasperini et al. (2018)	CAF	CTG	AUTO	Autologous	None	‐	None	NR	NR	PD, RD, CAL, WKG, CRC%
Çetiner et al. (2018)	CAF	PRP	BIO	Curasan, Pharma Gmbh AG, Lindigstrab, Germany	ADM	ALLO	SureDerm, Seoul, Korea	NR	NR	RD, RW, WKG, PI, GI, PD, CAL, RC%
Abou‐Arraj et al. (2017)	CAF	ADM‐A	ALLO	AlloDerm BioHorizons	ADM‐B	ALLO	Puros Dermis Zimmer Biomet	NR	NR	WKG, RD, GT
Jepsen et al. (2017	CAF	CMX	XENO	NR	None	‐	None	NR	NR	RD, CRC%, RD, WKG, GT, CAL, PD
Cairo et al. (2016)	CAF	CTG	AUTO	Autologous	None	‐	None	NR	NR	RD, CRC%, PD, CAL, WKG, GT
McGuire and Scheyer (2016)	CAF	CMX	XENO	NR	CTG	AUTO	None	NR	NR	RD, CRC%,WKG, PD and CAL
Godavarthi et al. (2016	CAF	PPG	BIO	Autologous	ADM	ALLO	Alloderm Biohorizons	NR	NR	RD, CRC%, WKG, PD, CAL
Stefanini et al. (2016)	CAF	CMX	XENO	Geistlich Mucograft®, Geistlich Pharma AG	None	‐	None	NR	NR	RD,RW, WKG,GT,CAL,PD
Lops et al. (2015)	CAF	CTG	AUTO	Autologous	None	‐	None	NR	NR	RD, WKG,PD,CAL
Cairo et al. (2015)	CAF	CTG	AUTO	Autologous	None	‐	None	NR	NR	RD,WKG,PD,CAL
Milinkovic et al. (2015)	CAF	AFCC	AUTO	Autologous	CTG	AUTO	Autologous	NR	NR	RD, WKG, CAL
McGuire et al. (2014)	CAF	rhPDGF‐BB	BIO	GEM21‐S	CTG	AUTO	Autologous	NR	NR	RD,CAL,PD, WKG,RC%,CRC%, Pain intensity
Ahmedbeyli et al. (2014)	CAF	ADM	ALLO	AlloDerm, BioHorizon, USA	None	‐	None	NR	NR	RD, WKG, GT
Zucchelli et al. (2014)	CAF	CTG	AUTO	Autologous	None	‐	None	29.8 ± 3.2	40.2 ± 6.8	RD, CRC%, PD, CAL, WKG, Pain Intensity
Cardaropoli et al. (2014)	Modified CAF	CMX	XENO	Mucograft, Geistlich	None	‐	None	NR	NR	RD, PD, CAL, WKG,GT
Alkan and Parlar (2013)	CAF	EMD	XENO	Emdogain®, Institut Straumann AG, Basel, Switzerland	CTG	AUTO	Autologous	NR	NR	RD,RW, PD, CAL, RC%, WKG,GT
Kuis et al. (2013)	CAF	CTG	AUTO	Autologous	None	‐	None	NR	NR	PI, FMBS,PD, RD, WKG,CAL,RC%,CRC%
Köseoğlu et al. (2013)	CAF	GF	AUTO	Autologous	CMX	XENO	Collagen AD	NR	NR	PI, GI, PD, CAL, RD, RW, WKG,GT
Roman et al. (2013)	CAF	EMD	XENO	Emdogain®, Institut Straumann AG, Basel, Switzerland	CTG	AUTO	Autologous	NR	NR	RD,RC%,CRC%,WKG
Kumar and Murthy (2013)	CAF	PCG	BIO	Autologous	CTG	AUTO	Autologous	NR	NR	PI, GI, PD, CAL, RD, Pain Intensity, WKG
Cordaro et al. (2012)	CAF	EMD	ALLO	Emdogain®, Institut Straumann AG, Basel, Switzerland	None	–	None	NR	NR	RD, PD, CAL,WKG,FMBS,PI
Cardaropoli et al. (2012	CAF	CMX	XENO	Mucograft, Geistlich	CTG	AUTO	Autologous	NR	NR	RD,PD, CAL, WKG
Alkan and Parlar (2011)	CAF	EMD	ALLO	Emdogain®, Institut Straumann AG, Basel, Switzerland	CTG	AUTO	Autologous	NR	NR	RD, CAL, PD
Jankovic et al. (2010)	CAF	EMD	ALLO	Emdogain®, Institut Straumann AG, Basel, Switzerland	PRF	BIO	Autologous	NR	NR	RD, PD, CAL, Pain Intensity
Del Pizzo et al. (2005)	CAF	EMD	ALLO	Emdogain Biora AB	None	–	None	NR	NR	RD, RW, PD, CAL,WKG
Spahr et al. (2005)	CAF	EMD	AUTO	Emdogain Biora AB	Placebo	–	PGA	NR	NR	RD,RW, WKG,CAL,PD
McGuire and Nunn (2003)	CAF	EMD	ALLO	Emdogain Biora AB	CTG	AUTO	Autologous	NR	NR	RD,RW, PD, CAL, WKG
Hägewald et al. (2002)	CAF	EMD	ALLO	Emdogain Biora AB	Placebo	–	PGA	NR	NR	RD,RW, WKG, CAL,PD
Zucchelli et al. (1998)	CAF	GTR	ALLP	NR	CTG	AUTO	Autologous	NR	NR	PD, CAL, RD,WKG,RC%

Abbreviations: CAF‐ Coronally Advanced Flap, mCAF‐ modified Coronally Advanced Flap, PCM‐ Platelet concentrate matrix, CMX‐ Xenogenic Collagen Matrix, CTG‐ Connective Tissue Graft, EMD‐ Enamel Matrix Derivative, HA‐ Hyaluronic Acid, ADM‐ Acellular Dermal Matrix, PRF‐ Platelet Rich Fibrin, PRP‐ Platelet Rich Plasma, ADMA‐ Acellular Dermal Matrix(AlloDerm BioHorizons), ADMB‐ Acellular Dermal Matrix(Puros Dermis Zimmer Biomet), PPG‐ Periosteal Pedicle Graft, AFCC‐ Autologous Fibroblast Cell Culture, rh‐PDGF‐BB – recombinant human Platelet Derived Growth Factor‐ BB, GF‐ Gingival Fibroblasts, PCG‐ Platelet Concentrate Graft, GTR(R)‐ Resorbable Guided Tissue Regeneration Membrane, GTR(NR)‐ None Resorbable Guided Tissue Regeneration Membrane, AUTO‐ Autologous Biomaterial, ALLO – Allogenic Biomaterial, XENO‐ Xenogenic Biomaterial, ALLP – Alloplastic Biomaterial, BIO‐ Biologic Biomaterial, RD‐ Recession Depth, RW‐ Recession Width, PD‐ Probing Depth, CAL‐ Clinical Attachment Level, WKG‐ Width of Keratinised Gingiva, RC% ‐ Percentage of Root Coverage, FMBS – Full Mouth Bleeding Score, CRC% ‐ Complete Root Coverage %, GT‐ Gingival Thickness, PI‐ Plaque index, GI‐ Gingival Index, BOP‐ Bleeding on Probing, RES‐ Aesthetic score, NR – Not Reported.

This systematic review analyzed a total of 1733 sites from 936 participants included in 39 RCTs. The trials included 546 females and 390 male participants and the age range of the participants recruited in the included RCTs were 18–74 years.

### 3.2 Network meta‐analysis

For the NMA, 19 RCTs with a total of 858 teeth in in the entire network was considered.

#### 3.2.1 Keratinized gingival width

**Figure 2:**
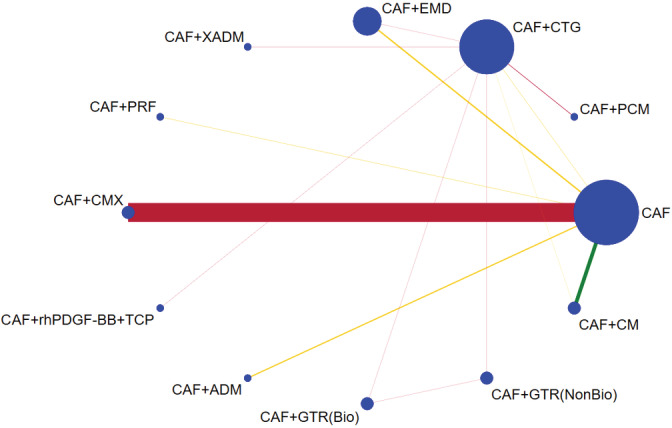
Network geometry plot for keratinized gingival width

**Figure 3:**
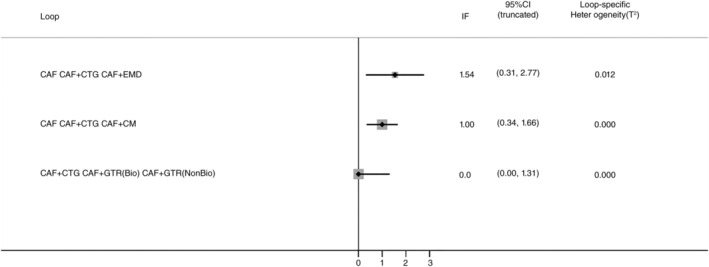
The inconsistency plot for KGW

**Figure 4:**
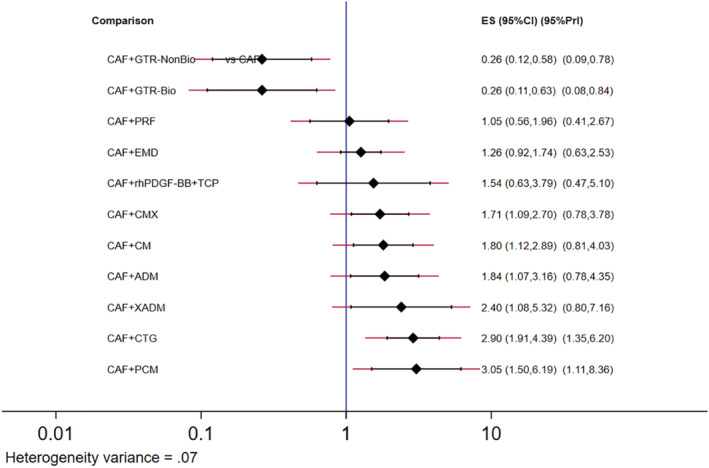
Predictive interval (Prl) and confidence interval plot (Crl) for KGW

**Figure 5:**
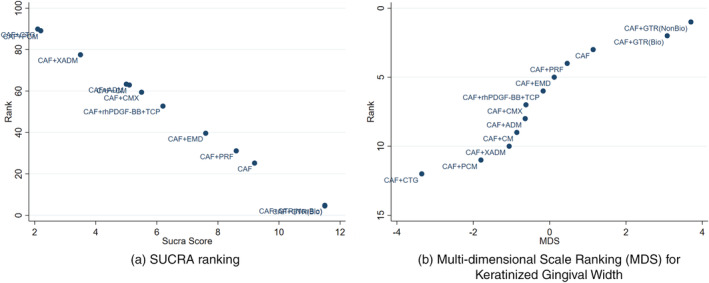
SUCRA ranking; 2E. Multi‐dimensional scale ranking (MDS) for keratinized gingival width

The most common comparator is between CAF, CAF + CMX, CAF + CTG and CAF + EMD. The risk of bias is found to be low between CAF and CAF + CM and high between other comparisons (Figures 2, 3, 4, 5). A total of *N* = 858 patients are included in the entire network. There are three studies included in CAF and CAF + CTG and four studies in CAF and CAF + EMD. The percentage contribution of each direct comparison in the entire network CAF and CAF + CTG (13.33%) followed by CAF and CAF + rhPDGF‐BB + TCP (10.33%) and that of each pairwise summary effect is from CAF and CAF + ADM (100%) followed by CAF and CAF + CTG (99.0%). CAF + GTR(NonBio) (11) and CAF + CMX (12) have significant variance and inconsistency. CAF + GTR‐Bio and CAF + GTR‐NonBio more likely to perform poor in future studies in gaining width thickness of gingival recession cases. A favorable outcome is expected through CAF + PCM and CAF + CTG in present (Crl) and future studies (Prl). CAF + CTG and CAF + PCM are ranked higher in SUCRA ranking. GTR Bio and Non‐Bio most distinct and dissimilar among other materials as illustrated in MDS rank.

#### 3.2.2 Percentage of root coverage

**Figure 6:**
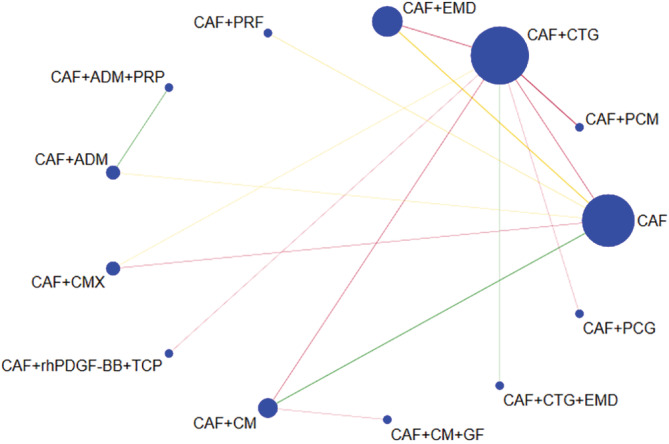
Network geometry plot for percentage of root coverage

**Figure 7:**
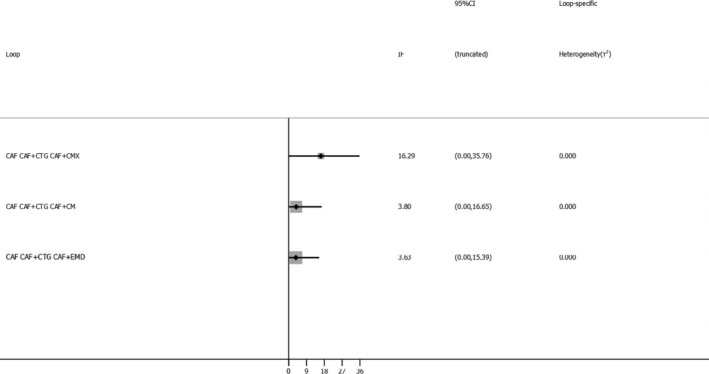
Inconsistency plots for Keratinized tissue width; Percentage of Root coverage and Recession Height

**Figure 8:**
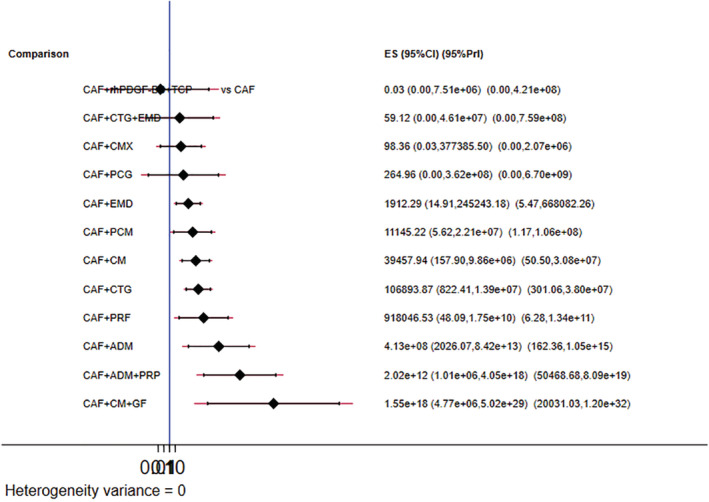
Predictive interval and confidence interval plot for percentage of root coverage

**Figure 9:**
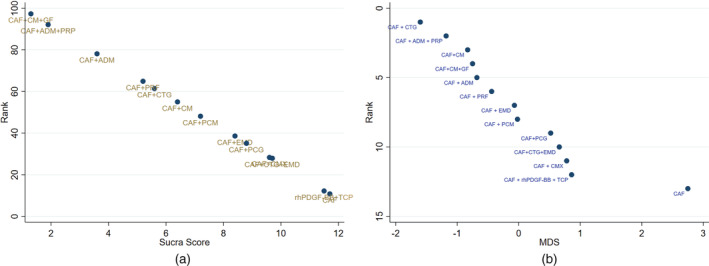
SUCRA ranking; 3E: Multi‐dimensional scale ranking (MDS) for percentage of root coverage

The network geometry plot illustrates the total number of subjects (*N* = 826) and comparisons between each intervention (Figures 6, 7, 8, 9). The number of RCT's in CAF and CTG are two and CAF + EMD are three. The majority of direct evidence contribution was by CAF + PCM and CAF + CTG (99.04) followed by CAF + ADM and CAF + CMX (98.36). The Majority of indirect evidence contribution is from CAF and CAF + CTG (41.15%) followed by CAF + CTG and CAF + PRF (20.54%). The evidence from entire network contribution is between CAF and CAF + ADM (9.87%) and CAF and CAF + CMX (8.34%).

There is no statistically significant inconsistency in the loop formed by CAF (01), CAF + PCM (03) and CAF + CMX (08) as the lower limit confidence intervals reaches the zero line. CAF + rhPDGF‐BB + TCP and CAF + CTG + EMD more likely to perform poor in future studies. It is predicted that CAF + CM + GF treatment would be 73.86% successful followed by CAF + ADM + PRP (45.83%).

CAF + CM + GF and CAF + ADM + PRP ranked higher in SUCRA ranking. Multidimensional scale (MDS) ranking shows that CAF + CTG and CAF + ADM + PRP are the most distinct interventions in improving the outcomes of root coverage in gingival recessions.

#### 3.2.3 Clinical attachment level

The highest ranked materials for this specific outcome in CAL were CAF + ADM and CAF + PRF in gingival tissue reconstruction. The effect estimate for CAF + ADM was 1.79(1.21, 2.84) and CAF + PRF was 1.43(0.99, 2.08) (Supplementary Figures 1(a)–(d)).

#### 3.2.4 Recession width

The highest ranked materials for RW gain in gingival recession were CAF + CTG and CAF + ADM. The effect estimate was 0.75(0.40, 1.39) and 0.37(0.11, 1.23) for CAF + CTG and CAF + ADM, respectively (Supplementary Figures 2(a)–(d)).

#### 3.2.5 Recession height

The highest ranked materials for RD gain were CAF + ADM and CAF + PCM. The effect estimate was found to be 2.03(1.38, 3.00) for CAF + ADM and 1.61 (1.04, 2.49) for CAF + PCM (Supplementary Figures 3(a)–(d)).

### 3.3 GRADE analysis

The overall level of evidence for the regenerative material is moderate. We predominantly downgraded the rating of the evidence due to different levels of risk of bias and imprecision (Table 4).

**Table 4:** GRADE analysis

Comparison	Network meta‐analysis (quality evidence)					Overall
	Pocket depth	Keratinized gingival width	Clinical attachment level	Recession width	Recession height	
CAF vs. CAF + PCM	Low	Low	Low	–	Low	Low
CAF vs. CAF + CTG	Low	Moderate	Low	Moderate	Low	Moderate
CAF vs. CAF + EMD	Low	Low	Moderate	Moderate	Low	Moderate
CAF vs. CAF + XADM	Low	Low	Low	Moderate	Low	Moderate
CAF vs. CAF + PRF	Low	Low	Moderate	Low	Low	Moderate
CAF vs. CAF + CMX	Moderate	Moderate	Moderate	Low	Low	Moderate
CAF vs. CAF + rhPDGF‐BB + TCP	Moderate	Moderate	Moderate	–	Moderate	Moderate
CAF vs. CAF + ADM	Moderate	Moderate	Moderate	–	Moderate	Moderate
CAF vs. CAF + GTR(Bio)	–	Moderate	–	–	–	Moderate
CAF vs. CAF + GTR(Non Bio)	–	Moderate	–	–	–	Moderate
CAF vs. CAF + CM	Moderate	Moderate	Moderate	–	Low	Moderate
**Overall**						Moderate

## 4 DISCUSSION

Our results from NMA explored the effectiveness of different biomaterials in periodontal regeneration (i.e., Gingival Recession). KGW and percentage of root coverage are considered as primary outcomes for validating the successful periodontal regeneration by a biomaterial (Lang & Löe, 1972). We found that, CAF + CTG ranked with highest probability followed by CAF + PCM in keratinised tissue width regeneration (Figure 5). A favorable outcome is expected through CAF + PCM and CAF + CTG as indicated from effect sizes, CrI and PrI. Similarly, CAF + CTG ranked with highest probability followed by CAF + ADM + PRP in favorable outcomes in percentage of root coverage. It is predicted that, CAF + CM + GF treatment would be 73.86% more successful followed by CAF + ADM + PRP (45.83%) (Figure 9).

**Figure 10:**
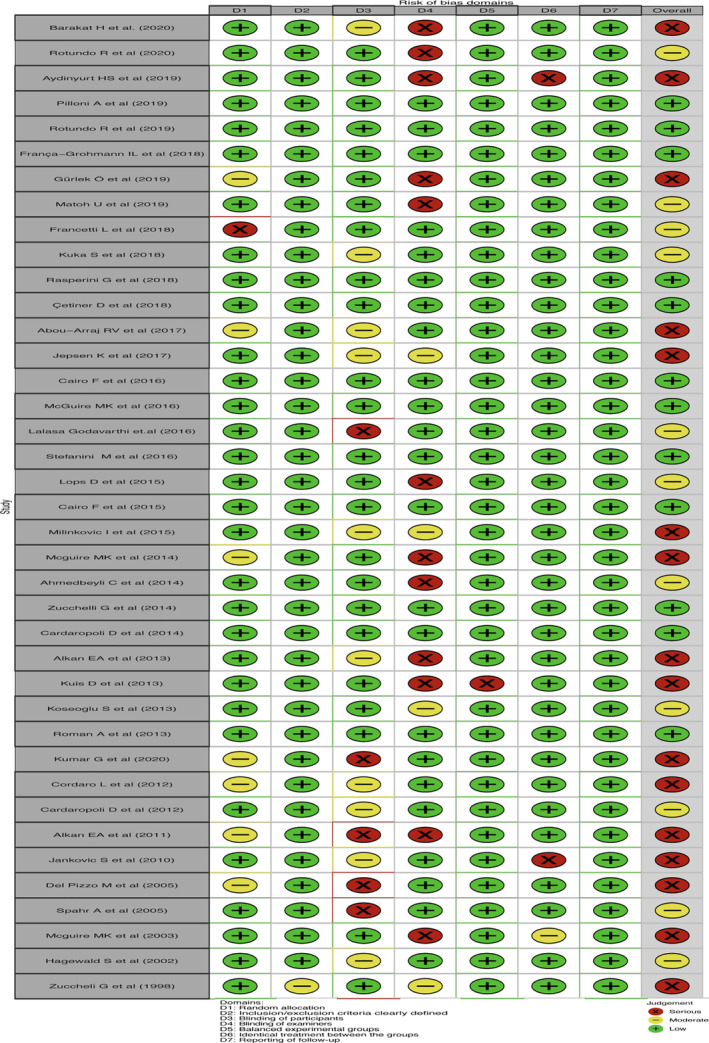
Risk of bias summary for all included studies

The ROB among studies that included CAF and CAF + CMX intervention was high and ROB between CAF and CAF + CM was low. Other studies were unclear or had moderate ROB in keratinised gingival width (KGW) outcome (Figure 1(a)). Similarly, ROB was high among majority of the comparisons except the comparison between CAF Vs CAF + CM for percentage of root coverage (Figure 2). The ROB for all the included studies was illustrated in Figure 10. According to the inconsistency plot, the loop formed by CAF, CAF + CTG, CAF + EMD, CAF + GTR (Nonbio) and CAF + CM had significant inconsistency in KGW outcome (Figure 1(b)). Similarly, CAF, CAF + PCM, CAF + CM, CAF + CTG + EMD and CAF + EMD had significant inconsistency in percentage of root coverage outcome (Figure 3). The overall evidence from all the comparisons for KGW and percentage of root coverage was found to be moderate (Table 3). The ranking of materials rated highest and lowest should be interpreted carefully by taking ROB and inconsistencies factors between these comparisons and dissimilarity between the materials illustrated by multidimensional scaling (Figure 1(e) and 5(b)).

CTG has always been considered as gold standard intervention for root coverage and for the modification of periodontal phenotype (Barootchi et al., 2020; Chambrone & Tatakis, 2015; Tatakis et al., 2015) because it demonstrates the best long‐term maintenance of treatment (Pini Prato, Franceschi, et al., 2018). However, it presents with limitations such as increased surgical morbidity, bleeding and postoperative pain. Therefore, clinicians and the patients look for alternatives that can meet the clinical need and also improve the post treatment quality of life (Moraschini et al., 2019). In contrast, the use of CTG substitutes does not affect postoperative pain and quality of life (Rotundo et al., 2019; Tonetti et al., 2018). For this reason, the decision making to choose among different biomaterial substitutes in adjunct to CAF for root coverage of single or multiple gingival recessions must be based on scientific evidence.

Recently, the effect of time on the stability of postsurgical results emerged as an important factor for root coverage and periodontal procedures (Cortellini et al., 2017; Pini Prato, Magnani, et al., 2018; Wu et al., 2017). A duration of 6 months is considered as a sufficient time for healing and tissue stability after mucogingival surgery (Cheng et al., 2007) and some authors have shown that the data obtained at this time can already predict the results of 3 years of the RCP (Cairo et al., 2015; Jepsen et al., 2017), and at 12 months the maturation of the tissue after the procedure is already complete (Gurtner et al., 2008; Smith et al., 2015).

A data on long term effects of different RCP has been recently being reported (Moslemi et al., 2011; Pini Prato et al., 2011; Pini Prato, Franceschi, et al., 2018; Pini Prato, Magnani, et al., 2018; Rasperini et al., 2018)and although there are still some controversies, CTG‐based techniques show the least changes over time (Pini Prato, Franceschi, et al., 2018; Rasperini et al., 2018). But, despite the fact that the evidence provides favorable results of early treatment (6 or 12 months) for gingival recessions (Francesco Cairo et al., 2014; Tavelli, Barootchi, et al., 2018), whether they persist for a longer time, has not yet been determined (Chambrone et al., 2019). In addition, a definitive conclusion cannot be drawn individually because of limited sample size and high drop outs (Chambrone et al., 2019; McGuire et al., 2012; Rasperini et al., 2018). Therefore, a time greater than 12 months, as a variable of the obtained treatment results, has never been explored. For this reason, this SR and NMA made direct and indirect comparisons between possible CTG substitutes with a minimum follow‐up time of 12 months, avoiding any influence of extrinsic factors in the healing process. Furthermore, the incorporation of an NMA can provide information on the effect of time on the changes that occur in the results obtained postoperatively and at the same time the different substitutes of the CTG are compared.

Although the treatment of choice, in terms of flap design, remains controversial (Santamaria et al., 2017; Zuhr et al., 2014), in order to guarantee homogeneity in the analyses of the present study, all included studies used the CAF as the flap design. All biomaterials had superior performance compared to CAF alone, for PD, KGW, CAL, RW, and RH parameters. These results are similar to those found by several systematic reviews aimed at evaluating the efficacy of RCP (Francesco Cairo et al., 2014; Roman et al., 2013; Tavelli, Asa'ad, et al., 2018).

One of the objectives of this article was to evaluate the effect of time on gingival recessions using the CAF as a flap design and comparing it with other biomaterials. Although it was found that the CAF + CTG and CAF + ADM + PRP approaches showed the best results in time for the percentage of root coverage, the CAF + CTG approach showed a greater difference in relation to the other approaches. These results are similar to those reported by other authors (Rasperini et al., 2018; Cairo et al., 2014; Dai et al., 2019) where they found that CAF + CTG have a tendency to displace gingival margin coronally, while CAF alone had a tendency towards apical relapse. It is reported that, due to biological filler content of CTG, it has the ability to adapt flap on the root surface (Francesco Cairo et al., 2016) and increases the marginal thickness of soft tissue. This enables greater chance of achieving root coverage (Rebele et al., 2014). This is fundamentally crucial also for the stability of the gingival margin, since an increase in the thickness of the gingival tissue after a CTG has been associated with the effect of progressive adhesion over the years (Pini‐Prato et al., 2010; Rasperini et al., 2018). Furthermore, it is also similar to that reported by Chambrone et al. (2019)) and Mehta et al. (2019)), where authors mention that there is, evidence suggesting that ADMs appear as the soft tissue surrogate that can provide the most similar results to those achieved by CTG for single or multiple recessions (Lee et al., 2002). On contrary, Leknes et al. (2005)) did not find any difference in time intervals between CAF and GTR for root coverage. It is suggested that, a strict oral hygiene maintenance after each appointment, after the root coverage procedure was recommended (McGuire et al., 2014; Pini Prato et al., 2011; Zucchelli et al., 2018).

The importance of at least 2 mm KTW has been demonstrated as an important factor for the stability of the gingival margin over time (Pini Prato, Magnani, et al., 2018). Furthermore, it has also been suggested that KTW plays a crucial role in facilitating long‐term maintenance of the patients themselves and reducing the risk of soft tissue relapse (Stefanini et al., 2018; Zucchelli et al., 2014). In our analysis, we found that KTW was a significant predictor that greatly affected treatment slopes, which is also mentioned by Tavelli, Barootchi, et al. (2019) and Tavelli, Ravidà, et al. (2019). Among treatment approaches, CAF + PCM exhibited positive slopes for KTW increase in future recessions. A possible explanation could be the potential of this material to increase the width of the keratinized tissue (Yu et al., 2018). Despite all this, our results confirm that a CTG was the best treatment to increase KTW over time.

This SR included only data from RCTs, analyzing the best available evidence where different biomaterials were used as a complement to CAF (PCM, EMD, XADM, PRF, CMX, rhPDGF‐BB + TCP, ADM, GTR (Bio), GTR (Non Bio), and CM). Furthermore, studies where there were smokers were not included, as smokers may have greater gingival margin instability than non‐smokers (Raes et al., 2015) due to ecological, immunological and vascular deficiencies caused by tobacco use (Palmer et al., 2005).

Some weaknesses of this study should be highlighted. First, most of the studies presented a high or moderate risk of bias, which increases the inconsistency of the results, leading to the fact that in the comparisons between CAF only with CAF with a biomaterial they presented a moderate GRADE analysis, thus decreasing, the recommendation of clinical results.

Another problem could be the differences in the process of making platelet concentrates. Variations in centrifuge type, speed, and G‐force could change membrane patterns and, consequently, the number of growth factors and cytokines (Miron et al., 2019). Furthermore, the limited research and high risk of bias in these studies, mentioned by Li et al. (2019)) and Moraschini and Barboza (2016)), can make the interpretation of the results difficult.

Among the limitations of the literature, we observed that included RCTs provided no information regarding gingival phenotype of the patient at the start of the study or at follow‐up intervals. Gingival phenotype was suggested to play a key role in determining future graft procedures, and this could not be explored with NMA. There was no significant added information from the analysis about gingival thickness (i.e., gingival thickness ≥ 0.8 mm or 1.2 mm has shown to be associated with greater chance of complete root coverage) (Baldi et al., 1999; Cairo et al., 2016). In addition, due to the lack of individual patient data, the impact of age and gender on the stability of the results was not investigated. However, in a recent article, age and gender were not found to be relevant factors in maintaining the stability of the gingival margin (Rasperini et al., 2018).

## 5 IMPLICATIONS FOR CLINICAL PRACTICE AND FUTURE DIRECTIONS

Our SR and NMA found that ADM + PRP and PCM have the better clinical performance as an adjunct to CAF, for the percentage of root coverage and KTW, respectively, in the treatment of Miller's class I and II gingival recessions (Cairo RT I). Based on the ranking of biomaterials, clinician will be able to make informed decisions in daily clinical practice. Standardization of the methods for using these biomaterials is essential to ensure that results are reproducible and predictable for monitoring long‐term tissue stability and behavior.

## 6 CONCLUSION

Our NMA found that CAF + CTG and CAF + PCM for KGW and CAF + CM + GF AND CAF + ADM + PRP for percentage of root coverage were ranked higher and would perform better in future clinical studies. The highest ranked material in improving CAL was CAF + ADM and CAF + PRF. In conclusion, CTG, ADM, and CM along with GFs showed improved stability for ≥12 months follow‐up, when used in adjunct to CAF in terms of better percentage of root coverage and improved KGW. However, the overall evidence was moderate and therefore, well designed clinical trials are needed.

## CONFLICT OF INTEREST

The authors declare no conflicts of interest related to the contents of this study.

## AUTHORS CONTRIBUTION

*Concepts, design, definition of intellectual content, literature search, clinical studies, experimental studies, data acquisition, data analysis, statistical analysis, manuscript preparation, manuscript editing, manuscript review, and guarantor*: Sourav Panda, Shahnawaz Khijmatgar, Heber Arbildo‐Vega, Abhaya Chandra Das, Manoj Kumar, Mohit Das, Leonardo Mancini, and Massimo Del Fabbro.

## SOURCES OF FUNDING STATEMENT

No external funding, apart from the support of the authors' institution, was available for this study.

## Supporting information


**Supplementary Figure 1a** Network geometry plot for clinical attachment level

**Supplementary Figure 1b** Inconsistency plot for clinical attachment level

**Supplementary Figure 1c** Predictive interval and confidence interval plot for clinical attachment level

**Supplementary Figure 1d** SUCRA and multidimensional ranking for clinical attachment level

**Supplementary Figure 2a** Network geometry plot for recession width

**Supplementary Figure 2b** Predictive interval plot for recession width

**Supplementary Figure 2c** SUCRA and multidimensional scale ranking for recession width

**Supplementary Figure 3a** Network geometry plot for recession height

**Supplementary Figure 3b** Inconsistency plot for recession height

**Supplementary Figure 3c** Predictive interval plot for recession height

**Supplementary Figure 3d** SUCRA and multidimensional scale ranking for recession height

**Appendix** **S1:** Supporting information

## Data Availability

Data sharing not applicable ‐ no new data generated, or the article describes entirely theoretical research.
