# Supplementary figures and images for: Stability of biomaterials used in adjunct to coronally advanced flap: A systematic review and network meta‐analysis
Source: Clin Exp Dent Res. 2021 Nov 29;8(1):421–38. doi: 10.1002/cre2.461 (PMC8874057; doi:10.1002/cre2.461)

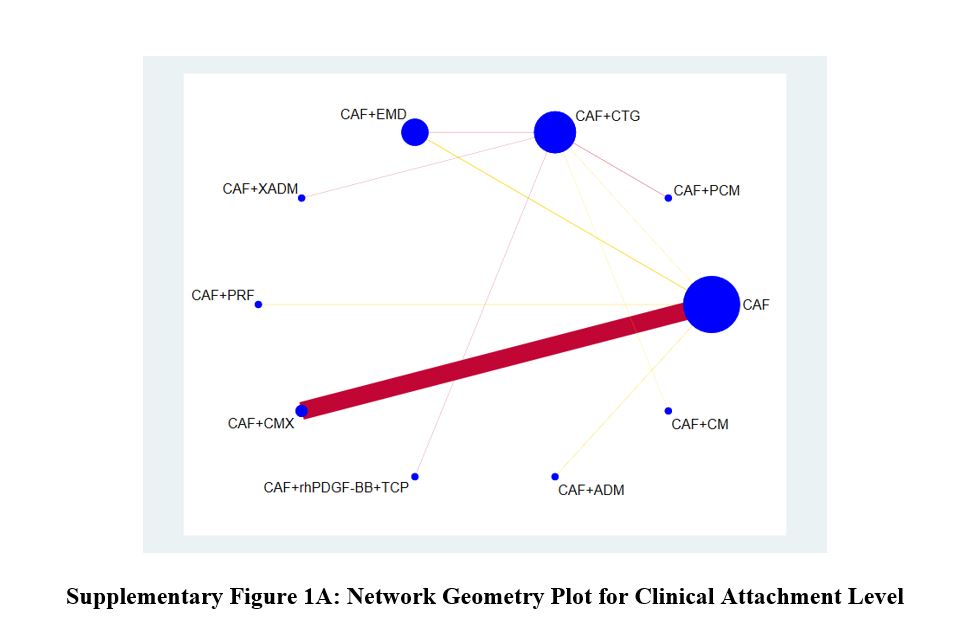

Supplement: Supplementary file 1 — Supplementary Figure 1a Network geometry plot for clinical attachment level [file CRE2-8-421-s007.JPG]

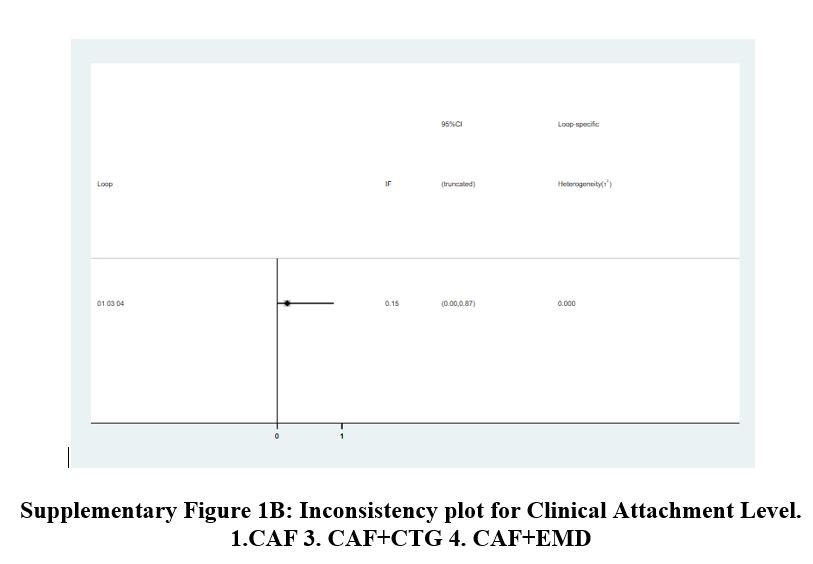

Supplement: Supplementary file 2 — Supplementary Figure 1b Inconsistency plot for clinical attachment level [file CRE2-8-421-s002.JPG]

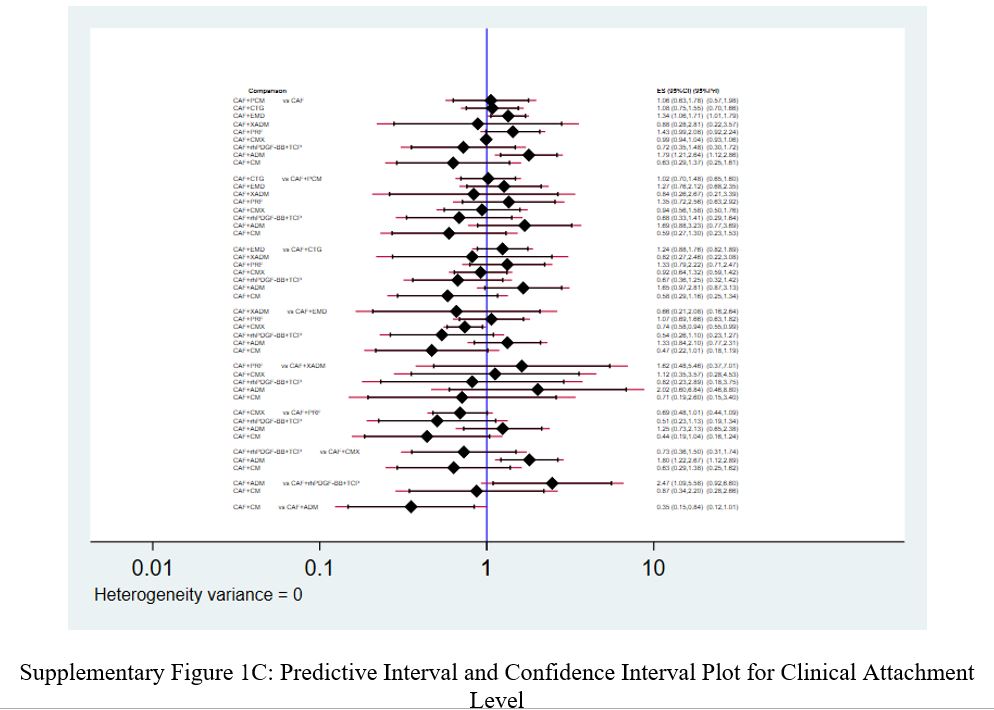

Supplement: Supplementary file 3 — Supplementary Figure 1c Predictive interval and confidence interval plot for clinical attachment level [file CRE2-8-421-s011.JPG]

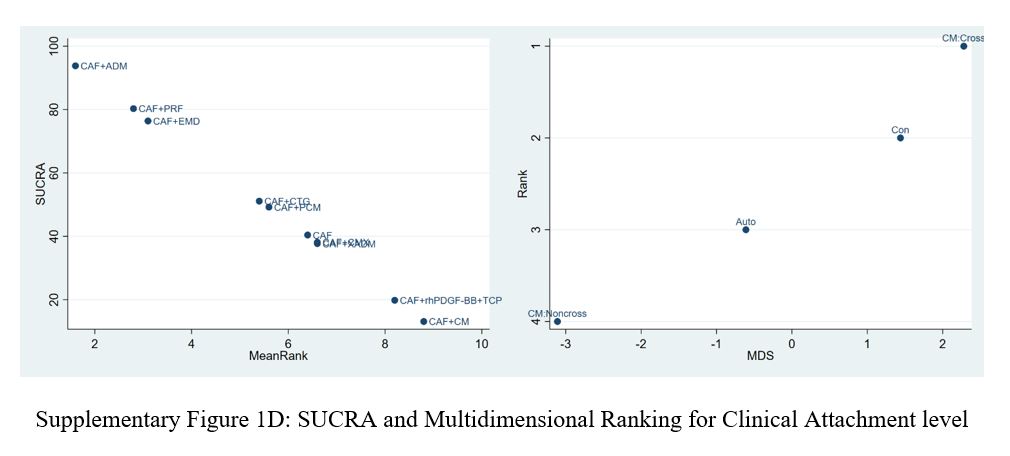

Supplement: Supplementary file 4 — Supplementary Figure 1d SUCRA and multidimensional ranking for clinical attachment level [file CRE2-8-421-s010.JPG]

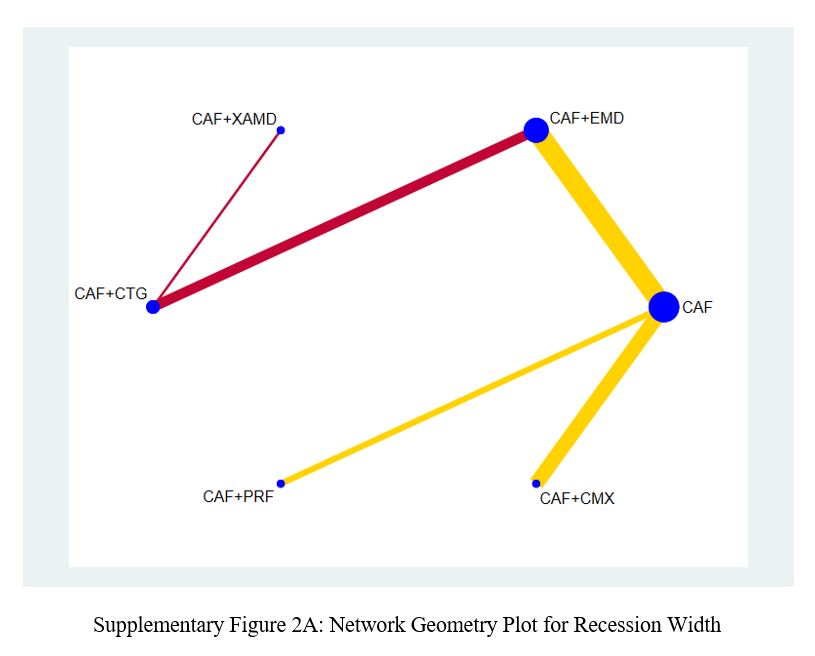

Supplement: Supplementary file 5 — Supplementary Figure 2a Network geometry plot for recession width [file CRE2-8-421-s008.JPG]

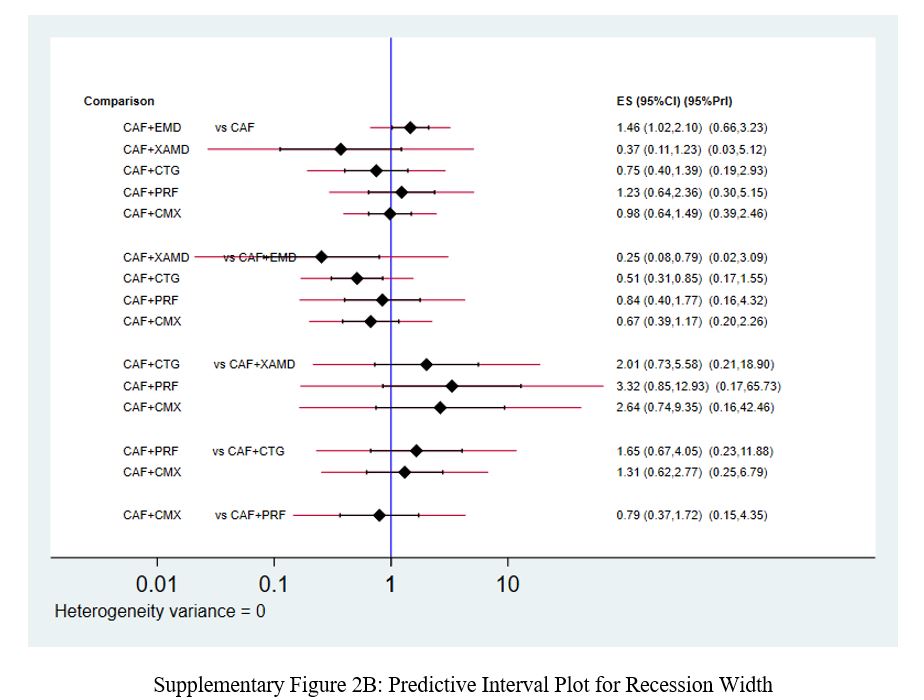

Supplement: Supplementary file 6 — Supplementary Figure 2b Predictive interval plot for recession width [file CRE2-8-421-s003.JPG]

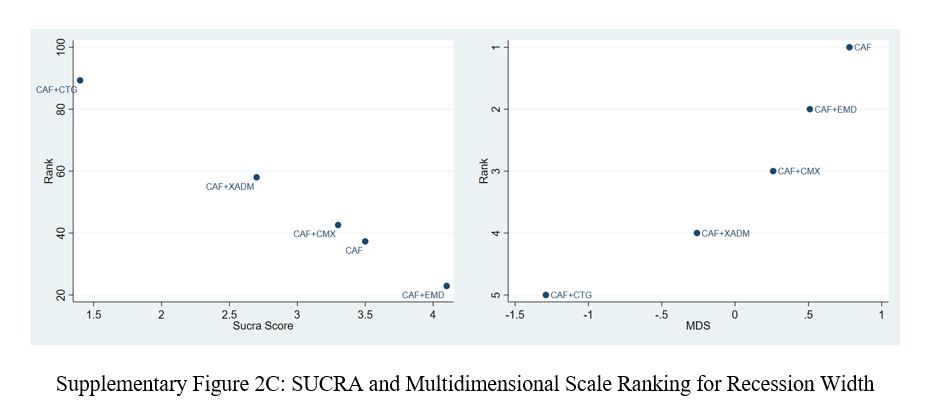

Supplement: Supplementary file 7 — Supplementary Figure 2c SUCRA and multidimensional scale ranking for recession width [file CRE2-8-421-s001.JPG]

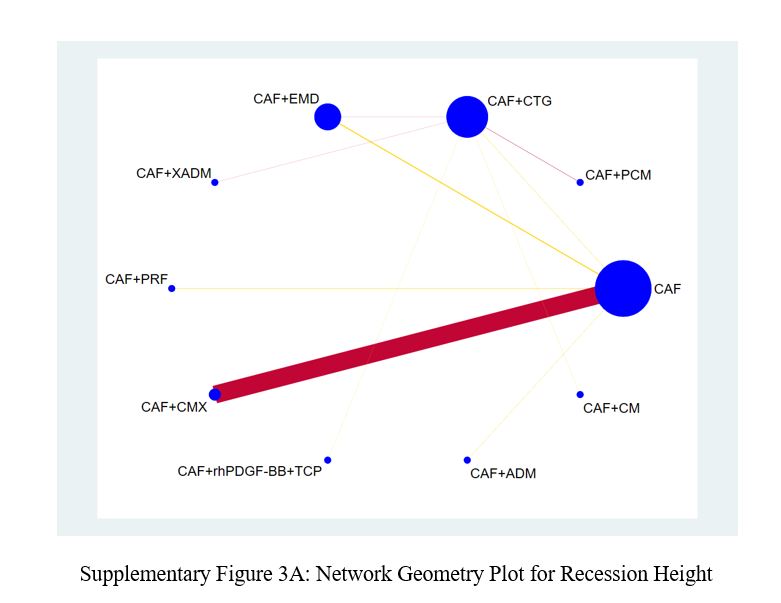

Supplement: Supplementary file 8 — Supplementary Figure 3a Network geometry plot for recession height [file CRE2-8-421-s006.JPG]

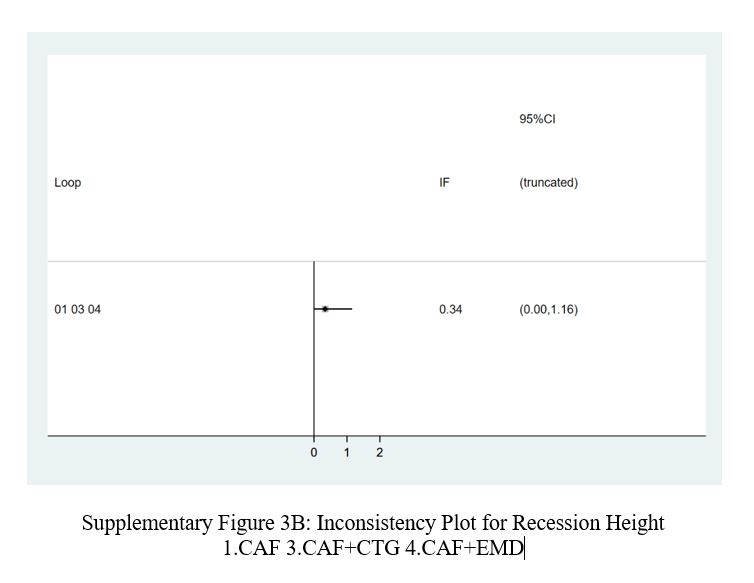

Supplement: Supplementary file 9 — Supplementary Figure 3b Inconsistency plot for recession height [file CRE2-8-421-s005.JPG]

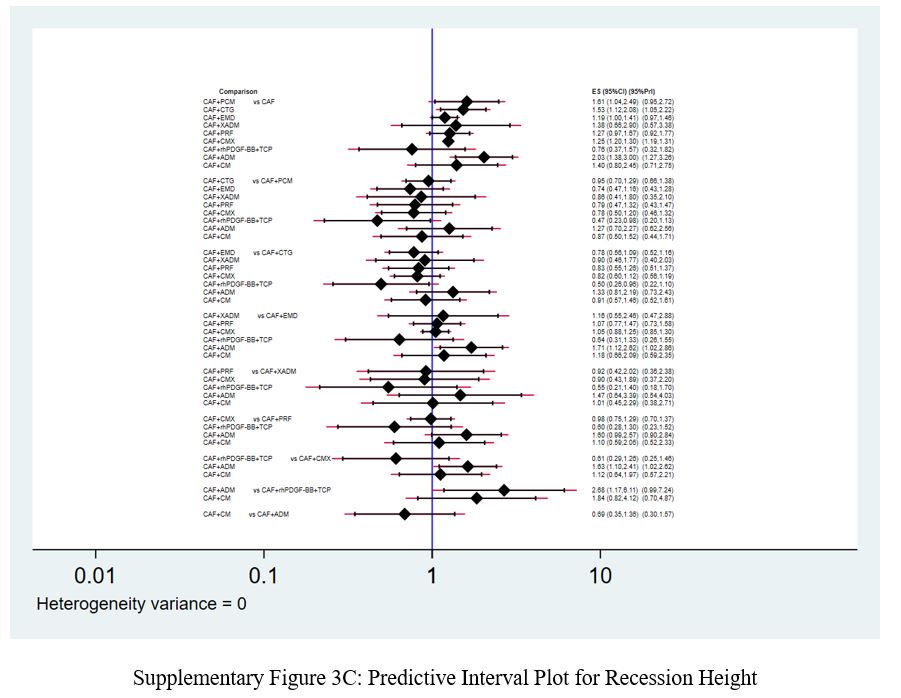

Supplement: Supplementary file 10 — Supplementary Figure 3c Predictive interval plot for recession height [file CRE2-8-421-s004.JPG]

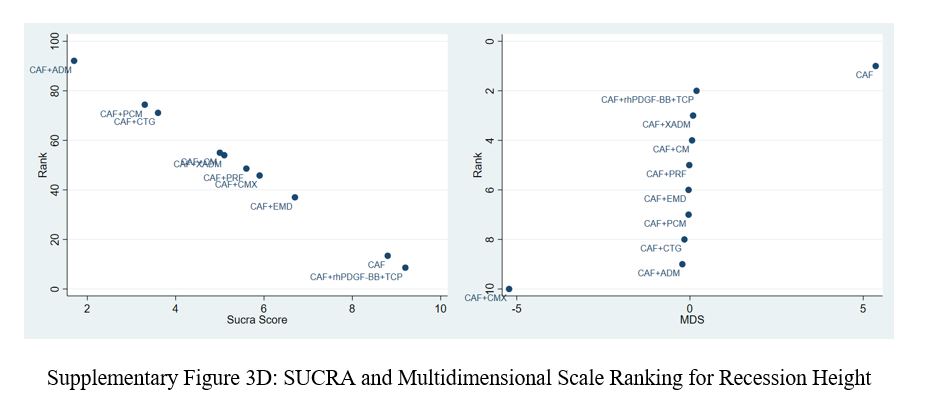

Supplement: Supplementary file 11 — Supplementary Figure 3d SUCRA and multidimensional scale ranking for recession height [file CRE2-8-421-s012.JPG]
