# Supplementary material for: Stability of biomaterials used in adjunct to coronally advanced flap: A systematic review and network meta‐analysis
Source: Clin Exp Dent Res. 2021 Nov 29;8(1):421–38. doi: 10.1002/cre2.461 (PMC8874057; doi:10.1002/cre2.461)
Supplement: Supplementary file 12 — Appendix S1: Supporting information [file CRE2-8-421-s009.pdf]

Author	Year	Single/Multiple recession	CAF Technique / modifications	Procedure	Category of Material	Biomaterial	N Treated	N Analysed	Biomaterial Trade Name	Outcomes assessed	Keratinized Gingival Width						Clinical Attachment Level					
											Pre (Mean)	SD	Post (Mean)	SD	Change	SD	Pre	SD	Post	SD	Change	SD
Barakat H, Dayoub S.	2020	Single	CAF	CAF + PCM		PCM	22	20	Autologo	RD, PD, CAL, WKG, RC%, CRC%, esthetic satisfaction and pain assessments	2.17	0.65	3.53	0.82	1.35	0.4	3.9	0.87	1.85	0.65	-2.05	0.58
				CAF + CTG		CTG	22	20	Mucograft collagen matrix; Geistlich Pharma		2.2	0.61	3.5	0.65	1.3	0.44	3.62	1.02	1.55	0.6	-2.07	0.63
Rotundo R, Genzano L, Nieri M, Covani U, Peñarrocha-Oltra D,	2020	Multiple	CAF	CAF + CMX		CMX	12	12	Geistlich Mucograft®, Geistlich Pharma AG	Esthetic condition and the overall satisfaction	3.3	1.5	NR		NR		3.8	0.6	NR		NR	
				CAF	Control	None	12	12	None		3.5	1.8	NR		NR		4.2	1.3	NR		NR	
Aydinyurt HS, Tekin Y, Ertugrul AS.	2019	Single	CAF	CAF + SCTG + EMD		EMD	19	19	Emdogain®, Straumann, Basel, Switzerland	Recession depth/width, root coverage percentage and root coverage aesthetic score (RES)	NR		NR		NR		NR		NR		NR	
				CAF + SCTG		SCTG	19	19	Autologo		NR		NR		NR		NR		NR		NR	
Pilloni A, Schmidlin PR, Sahrmann P, Sculean A, Rojas MA.	2019	Single	CAF	CAF + HA		HA	15	15	HyaDENT BG, Bioscience, Germany	Rec reduction, CAL gain, changes in PPD, width of KT, CRC, mean RC, pain intensity, discomfort, and	NR		NR		NR		NR		NR		NR	
				CAF	Control	None	15	15	None		NR		NR		NR		NR		NR		NR	
Rotundo R, Genzano L, Patel D, D'Aiuto F, Nieri M.	2019	Multiple	CAF	CAF + CMX		CMX	12	12	Geistlich Mucograft®, Geistlich Pharma AG	Rec depth, Rec width, BOP, PI, PD, CAL, KT, KT thick	3.3	1.5	2.7	1.2	NR		3.8	0.6	1.7	0.6	NR	
				CAF	control	None	12	12	None		3.5	1.8	2.5	1.1	NR		4.2	1.3	1.9	1	NR	
França-Grohmann IL, Menck JP, Rocha M, Viana RC, Gonzáles K,	2019	Single	CAF semilunar	CAF + EMD		EMD	15	15	Emdogain®, Institut Straumann AG, Basel, Switzerland	RH, RW, WKT, TKT, PD and CAL	3.32	0.71	3.51	0.77	0.19	0.57	3.9	0.42	1.6	0.74	− 2.30	0.67
				CAF	Control	None	15	15	None		3.23	0.88	3.33	0.79	0.1	0.35	3.99	0.57	1.87	0.72	− 2.11	0.65
Gürlek Ö, Gümüs P, Nizam N, Buduneli N.	2019	Multiple	Modified CAF	CAF + XADM		XADM	15	12	Mucoderm, Botiss Gmbh, Berlin, Germany	RD, RW, KTW, PD and CAL	3.4	1.2	3.7	0.93	0.32	0.52	4.4	1.1	0.71	1.3	−3.70	1.6
				CAF + CTG		CTG	15	12	Autologo		3.7	1.1	4.2	0.98	0.51	0.6	4.4	1	0.49	0.98	−3.90	1.1
Matoh U, Petelin M, Gašperšič R.	2019	Single	CAF	CAF + CM		CM	10	10	OsteoBiol Derma, Tecnoss	RD, PD, CAL, KTH, GT and RC%	NR		NR		NR		3.2	0.7	NR		NR	
				CAF + CTG		CTG	10	10	Autologo		NR		NR		NR		3.7	1.2	NR		NR	
Francetti L, Weinstein R, Taschieri S, Corbella S.	2018	Single	CAF	CAF + CTG	Test	CTG	10	10	Autologo	REC, CRC, KG, CAL and RC%	2.3	0.82	3	0.82	NR		4.11	0.78	2.05	0.96	NR	
				CAF	Control	None	10	10	None		2.89	1.05	2.89	0.78	NR		4.22	0.83	2.78	1.39	NR	
Kuka S, Dirikan S, Cakar G, Yilmaz S.	2018	Multiple	CAF	CAF + PRF		PRF	12	12	3000 rpm for 10 min	RH, RW, PD, CAL, GT, KT, PI, GI and BOP	2.6	0.77	3.3	0.98	0.7	0.42	4.25	0.35	2.15	0.78	−2.10	0.61
				CAF	Control	None	12	12	None		2.95	1.01	3.6	1.29	0.65	0.47	4.54	0.3	2.8	0.35	−1.74	0.24
Rasperini G, Acunzo R, Pagni R, Tonetti M, Pini	2018	Single	CAF	CAF + CTG		CTG	12	12	Autologo	PD, REC, CAL, KT and CRC	3.2	1	4.8	0.7	NR		3.7	0.8	2	0.7	NR	
				CAF	Control	None	13	13	None		3.8	1.5	3.6	0.7	NR		3.5	1	2.4	0.9	NR	
Çetiner D, Gökalp P, Özdemir B, Turgut Z.	2018	Multiple	CAF	CAF + ADM + PRP		PRP	14	12	Curasan, Pharma Gmbh AG, Lindigstrab, Germany	GRD, GRW, WKT, PI, GI, PD, CAL, CRA and RC%	1.2	0.3	3.8	0.5	NR		5	0.4	2.3	0.2	NR	
				CAF + ADM		ADM	14	12	SureDerm, Seoul, Korea		1.2	0.4	3.6	0.5	NR		4.8	0.4	2.7	0.4	NR	
Abou-Arraj RV, Kaur M, Vassilopoulos PJ, Geurs	2017	Single	CAF	CAF + ADM-A		ADM-A	10	8	AlloDerm BioHorizons	KG, RD and GT	3.1	3.1	NR		0.99		4.4		1.94		NR	
				CAF + ADM-B		ADM-B	10	9	Puros Dermis Zimmer Biomet		3.3	3.3	NR		0.91		3.8		1.52		NR	
Jepsen K, Stefanini M, Sanz M, Zucchelli G,	2017	Single	CAF	CAF + CMX		CMX	18	18	NR	RC, CRC, REC, KT, GT, CAL and PD	2.14	1.21	4.06	1.55	1.92	1	4.58	0.97	1.42	0.6	−3.17	1.11
				CAF	Control	None	18	18	None		2.22	1.39	3.25	0.81	1.03	1.1	4.61	0.87	1.94	0.8	−2.67	1.14
Cairo F, Cortellini P, Pilloni A, Nieri M,	2016	Multiple	CAF	CAF + CTG		CTG	16	16	Autologo	Rec, CRC, PD, CAL, KT and GT	2.8	0.8	4.7	0.9	1.8	0.6	4.2	0.8	1.2	0.4	NR	
				CAF	Control	None	16	16	None		3.1 ± 1	1	2.7	1.2	−0.4	0.9	4	0.9	1.6	0.6	NR	
McGuire MK, Scheyer ET.	2016	Single	CAF	CAF + CMX		CMX	17	17	NR	RC, CRC, KT, PD and CAL	2.44	1.02	3.41	1.06	NR		4.4	0.61	2.35	0.96	NR	
				CAF + CTG		CTG	17	17	Autologo		2.78	1.35	4.12	0.88	NR		4.5	0.61	1.65	0.7	NR	
Lalasa Godavarthi et.al	2016	Single	CAF	PPG+CAF		PPG	7	7	Autologo	RC, CRC, KT, PD and CAL					2.82	0.46					3.5	0.73
				ADMG+CAF		ADM	7	7	Allograft						1.61	0.56					3.39	0.63
Martina Stefanini at.al 2016	2016	Multiple	CAF	CAF		CAF	45	41	Autologo	Root Coverage	2	1.22	2.64	1.1	-0.64	0.12	4.82	1.09	2.11	1.23	2.71	0.14
				CAF+CMX		CAF+CMX	45	41	Auto+Xeno CM		1.97	1.33	3.02	1.26	1.05	0.07	4.79	1.01	2.09	1.1	2.7	0.09
Diego Lops et.al2015	2015	Single	CAF	CAF		CAF	14	13	Autologo	Gingival Recession	2.6	2.5-2.7	3	2.9-3.0							4.5	3.9-4.8
				CAF+CTG		CAF+CTG	14	12	Combination Auto		2.5	2.4-2.6	2.8	2.7-2.9							4.5	3.9-4.7
Fracesco Cairo et.al 2015	2015	Single	CAF	CAF		CAF	15	11	Autologo	Gingival Recession					2.8	0.8					2.2	0.8
				CAF+CTG		CAF+CTG	15	13	Combination Auto						4	0.8					2.2	1
						CAF	15	11	Autologo	Gingival Recession					2.5	1.3					2.3	0.9
						CAF+CTG	15	13	Combination Auto						4.5	1.3					2.3	0.9
Milinkovic	2015	Both	CAF	CAF +AFCC		AFCC	36	36	Autologo	Recession coverage, keratinized tissue width,clinical attachment level, healing	1.05	0.38	2.79	0.17	NR		4.53	0.26	1.53	0.18	NR	
				CAF +CTG		CTG			Autologo		1.11	0.38	3.37	0.22	NR		4.7	0.33	1.45	0.16	NR	
				CAF + rhPDGF-BB + TCP		rhPDGF- BB			GEM21-S		2.03	0.68	3.03	1.16	1	0.92	4.65	0.67	2.7	1.63	-1.95	0.96
Mcguire MK	2014	Both	CAF	CAF + CTG	Test	CTG	60	40	Autologo	% RC, % pt with 100% RC, Root hype	2.05	0.89	3.68	1.94	1.63	1.05	4.78	0.79	2.43	1.83	-2.35	1.04
				CAF+ADM	Test	ADM			AlloDerm, BioHorizon, USA		2.48	0.5	3.69	0.54	1.21	0.23	4.41	0.42	1.67	0.65	2.75	0.54
Ahmedbeyli	2014	Multiple	CAF	CAF	Control	None	48	48	None	t satisfaction, recession height redu	2.58	0.71	3.19	0.92	0.6	0.36	4.35	0.34	2.18	0.86	2.17	0.81
				CAF+CTG		CTG			Autologo		1.47		3.18		NR		4.19		1.32		NR	
Zuccheli	2014	Multiple	CAF	CAF	Control	None	50	50	None	C, PD, CAL, KTH, VAS, colour match,	1.43		2.75		NR		4.15		1.36		NR	
				mCAF+CM		CMX			Mucograft, Geistlich		1.89	0.99	2.96	0.76	1.07		3.57	0.95	1.34	0.62	-2.23	
Cardaropoli et al	2014	Multiple	Modified CAF	mCAF	Control	None	113	113	None	Depth (RD), PD, CAL, WKT, gingival t	1.91	1.01	2.61	1.08	0.7		3.49	1.07	1.61	0.82	-1.88	
				CAF+EMD		EMD			Emdogain		1.64	0.78	4.5	0.96	NR		5	1.25	1.54	0.8	NR	
Alkan and Parlar et al	2013	Multiple	CAF	CAF+CTG		CTG	56	56	Autologo	PD CAL, Percentage Root Coverage,	1.68	0.82	4.61	0.96	NR		4.68	0.98	1.38	0.78	NR	
				CAF+CTG		CTG			Autologo		1.33	1.17	2.7	0.6	NR		3.79	0.77	1.43	0.61	NR	
Kuis et al	2013	Single	CAF	CAF	Control	None	114	114	None	Bleeding Score(FMBS), PD, Gingival	1.33	1.19	2.25	0.76	NR						NR	
				CAF+CM+GF		CMX+GF			Autologo		3.27	1.34	2.86	1.05	NR		2.83	0.57	1.66	0.62	NR	
Koseoglu et al	2013	Single	CAF	CAF +CM		CMX	22	22	Collagen AD	ecession Width, WKT, Gingival Thick	2.95	1.01	2.59	0.97	NR		2.96	0.73	2	0.8	NR	
				CAF+CTG+EMD		CTG+EMD			Emdogain		2.43	1.27	3.75	1.01	NR		NR					
Roman et al	2013	Both	CAF	CAF+CTG		CTG	42	38	Autologo	Root Coverage Rate, Creeping Attac	2.38	0.98	3.7	0.77	NR		NR					
				CAF+PCG		PCG			Autologo		NR	NR	0.71	0.04	NR		NR	NR	1.92	0.16	NR	
Kumar&Murthy et al	2013	Single	CAF	CAF+CTG		CTG	24	24	Autologo	Recession Depth, VAS, Width of Ke	NR	NR	2.63	0.3	NR		NR	NR	2.42	0.41	NR	
				CAF+EMD		EMD			emdogain,straumann		2.72	2.78	3.09	0.57	0.36	0.44	4.64	1.31	2.05	0.76	2.52	0.93
Cordaro et al	2012	Multiple	CAF	CAF	Control	None	58	58	None	AL, Keratinised gingival width, bleed	2.78	0.65	3.19	0.59	0.41	0.42	4.34	1.13	1.55	0.52	2.17	0.84
				CAF+CM		CM			Mucograft, Geistlich		2.23	0.56	3.45	0.85	1.23	0.61	4.45	0.61	2.05	0.76	-2.41	0.83
Cardaropoli et al	2012	Multiple	CAF	CAF+CTG		CTG	22	22	Autologo	on depth,PD, CAL, keratinised gingiv	2.05	0.82	3.32	0.7	1.27	0.65	4.5	0.87	1.55	0.52	-2.95	0.82
				CAF+EMD		EMD			Emdogain		2.42	0.79	5.08	1.51	-2.67	1.37	5.04	0.64	1.35	0.48	3.69	0.68
Alkan and Parlar et al	2011	Single	CAF	CAF+CTG		CTG	24	24	Autologo	Recession depth, CAL, PD,	2.17	1.03	4.58	1.38	-2.42	1.44	5.08	0.48	1.55	0.28	3.53	0.38
				CAF+EMD		EMD			dogain, Straumann, Basel, Switzerl		1.3	0.56	1.9	0.81	NR		NR					
Jankovic et al	2010	Single	CAF	CAF+PRF		PRF	40	40	Autologo	ecession Depth, PD, CAL, Healing in	1.45	0.86	1.62	0.28	NR		NR					
				CAF+EMD		EMD			Emdogain Biora AB		1.47	0.74	2.47	0.52	NR		5.13	0.64	1.4	0.74	NR	
Pizzo et al	2005	Single	CAF	CAF	Control	None	60	60	None	ssion width, Probing depth, CAL, ke	1.67	0.82	2.13	0.52	NR		5.13	0.74	1.6	0.83	NR	
				CAF+EMD		EMD			Emdogain Biora AB	recession depth (RD), recession	2.083	0.986			0.65	0.993	5.22	0.819			3.19	1.17
Spahr et al	2005	Single	CAF	CAF+placebo	Control	Placebo	60	60	PGA	width (RW), height of keratinized	2,2	0.805			0.333	0.735	5.423	1.012			2.7	1.308
				CAF+EMD		EMD			Emdogain Biora AB		2.4	0.771	3.11		NR		6.15	1.226	1.59		NR	
Mcguire et al	2003	Single	CAF	CAF+CTG		CTG	40	40	Autologo	n width, Probing depth, CAL, kerati	2.5	0.889	4.06		NR		6.1	0.912	1.59		NR	
				CAF+EMD		EMD			Emdogain Biora AB	ecession depth	2.1	1			0.7	0.9	5.4	1			3.4	1.1
Hagewald et al	2002	Single	CAF	CAF+placebo	Control	Placebo	72	72	PGA	(RD), recession width (RW), height	2.4	1			0.3	0.9	5.5	1.2			3.1	1.3
				CAF+GTR(BIO)		GTR(BIO)			NR		1	0.9			1.7	1.6	7	0.8			4.9	0.6
				CAF+GTR(NonBIO)		GTR(NonBIO)			NR		1.1	0.4			1.7	1	7.1	0.6			4.7	1.1
Zuccheli et al	1998	Single	CAF	CAF+CTG		CTG	54	54	autologo	on depth, width of keratinised tissu	1	0.3			4.1	0.7	6.8	0.8			4.7	0.7

Author	Year	Percentage of root coverage						Recession Hieght						Recession Width					
		Pre	SD	Post	SD	Change	SD	Pre	SD	Post	SD	Change	SD	Pre	SD	Post	SD	Change	SD
Barakat H, Dayoub S.	2020	0		94.22	10.99%	94.22	10.99%	2.67	0.65	0.2	0.37	-2.47	0.47	NR		NR		NR	
		0		96.48	7.49%	96.48	7.49%	2.55	0.69	0.12	0.27	-2.42	0.52	NR		NR		NR	
Rotundo R, Genzano L, Nieri M, Covani U,	2020	NR		NR		NR		2.3	0.7	NR		NR		NR		NR		NR	
		NR		NR		NR		2.6	1	NR		NR		NR		NR		NR	
Aydinyurt HS, Tekin Y, Ertugrul AS.	2019	NR		NR		NR		3.91	1.2	1.34	1.11	NR		3.73	0.91	2	1.41	NR	
		NR		NR		NR		4.04	1.42	1.91	1.56	NR		3.73	0.75	2.21	1.62	NR	
Pilloni A, Schmidlin PR, Sahrmann P, Sculean A,	2019	NR		NR		NR		NR		NR		NR		NR		NR		NR	
		NR		NR		NR		NR		NR		NR		NR		NR		NR	
Rotundo R, Genzano L, Patel D, D'Aiuto F, Nieri	2019	NR		NR		NR		2.3	0.7	0.3	0.4	NR		3.2	0.7	0.7	1	NR	
		NR		NR		NR		2.6	1	0.6	0.3	NR		3.6	0.7	1.5	1.7	NR	
França-Grohmann IL, Menck JP, Rocha M,	2019	0		90.86	14.69	90.86	14.69	2.18	0.13	0.2	0.32	1.98	0.33	3	0.59	0.29	0.45	2.71	0.69
		0		79.76	17.44	79.76	17.44	2.32	0.21	0.47	0.4	1.85	0.41	3.08	0.46	0.64	0.58	− 2.44	0.52
Gürlek Ö, Gümüs P, Nizam N, Buduneli N.	2019	NR		NR		NR		2.7	1	0.32	0.52	2.4	0.92	3.1	0.71	0.9	1.5	2.2	1.5
		NR		NR		NR		2.6	0.77	0.12	0.33	2.5	0.75	3.1	0.88	0.22	0.61	2.9	1
Matoh U, Petelin M, Gašperšič R.	2019	0		85%	24	85%	24	2.2	0.7	0.4	0.7	NR		NR		NR		NR	
		0		100%		100%		2.6	1	0		NR		NR		NR		NR	
Francetti L, Weinstein R, Taschieri S, Corbella S.	2018	0		85.4	20.8	85.4	20.8	2.7	0.48	0.44	0.62	NR		NR		NR		NR	
		0		65.7	32.2	65.7	32.2	2.9	0.99	1.15	1.06	NR		NR		NR		NR	
Kuka S, Dirikan S, Cakar G, Yilmaz S.	2018	0		88.36	15.45	88.36	15.45	3.15	0.24	0.4	0.52	2.75	0.35	3.05	0.16	0.75	0.98	2.3	0.92
		0		74.63	8.05	74.63	8.05	3.36	0.34	0.85	0.24	2.51	0.33	3.39	0.73	1.25	0.49	2.09	0.69
Rasperini G, Acunzo R, Pagni R, Tonetti M, Pini	2018	NR		NR		NR		2.4	0.8	0.5	0.5	NR		3.8	0.8	NR		NR	
		NR		NR		NR		2.4	1	1	0.8	NR		3.8	0.6	NR		NR	
Çetiner D, Gökalp P, Özdemir B, Turgut Z.	2018	0		77.9	4.4	77.9	4.4	3.7	0.4	0.8	0.1	NR		3.8	0.3	0.8	0.2	NR	
		0		69.4	13.1	69.4	13.1	3.7	0.4	1.1	0.4	NR		3.8	0.3	1.1	0.3	NR	
Abou-Arraj RV, Kaur M, Vassilopoulos PJ, Geurs	2017	0		59.67%		59.67%		3.1		NR		−1.85		NR		NR		−2.52	
		0		83.70%		83.70%		2.7		NR		−2.29		NR		NR		−2.64	
Jepsen K, Stefanini M, Sanz M, Zucchelli G,	2017	0		9170.00%	12.05%	91.70%	12.05%	3.19	0.71	0.28	0.39	2.92	0.71	NR		NR		NR	
		0		8277.00%	17.05%	8277.00%	17.03%	3.11	0.78	0.58	0.6	2.53	0.72	NR		NR		NR	
Cairo F, Cortellini P, Pilloni A, Nieri M,	2016	NR		NR		NR		3.2	0.8	0.2	0.4	3	0.7	NR		NR		NR	
		NR		NR		NR		3	0.9	0.6	0.6	2.4	0.7	NR		NR		NR	
McGuire MK, Scheyer ET.	2016	0		77.6	29.2	77.6	29.2	NR		NR		NR		NR		NR		NR	
		0		95.5	12.8	95.5	12.8	NR		NR		NR		NR		NR		NR	
Lalasa Godavarthi et.al	2016					89												67.2	
						91.4												78.4	
Martina Stefanini at.al 2016	2016	75.05	26.24			1.22	18.44	3.34	1	0.93	1.1	2.41	0.1	4.1	0.93	2.01	1.81	2.09	0.88
		76.28	28.07					3.46	0.9	0.83	0.99	2.63	0.09	4.08	0.89	2.01	1.94	2.07	1.05
Diego Lops et.al2015	2015							3.1	2.9-3.3	1	0.9-1.1								
								2.9	2.8-3.0	0.5	0.5-0.6								
Fracesco Cairo et.al 2015	2015											2.5	1						
												2.9	0.8						
												2.4	0.9						
												2.8	0.9						
Milinkovic	2015	NR						3.41	0.17	0.37	0.09	NR		NR					
		NR						3.46	0.29	0.32	0.17	NR		NR					
Mcguire MK	2014			74.1	37.27	74.1	37.27	3.25	0.55	0.9	1.77	-2.35	1.22	NR					
				89.35	21.63	89.35	21.63	3.4	0.58	0.35	1.45	-3.05	0.87	NR					
Ahmedbeyli	2014			94.84	12.09	94.84	12.09	3.25	0.34	0.17	0.39	3.08	0.51	NR					
				74.99	28.07	74.99	28.07	3.21	0.26	0.83	0.94	2.37	0.83	NR					
Zuccheli	2014	NR						3.15		0.09		NR		NR					
		NR						3.05		0.3		NR		NR					
Cardaropoli et al	2014			93.25	10.01	93.25	10.01	2.48	0.94	0.2	0.34	-2.28		NR					
				81.49	23.45	81.49	23.45	2.43	1.07	0.58	0.87	-1.85		NR					
Alkan and Parlar et al	2013			89	17	89	17	3.54	1.14	0.5	0.79	NR		2.5	0.69	0.36	0.49	NR	
				93	17	93	17	3.29	0.71	0.25	0.59	NR		2.5	0.69	0.21	0.5	NR	
Kuis et al	2013			92.3	19.2	92.3	19.2	2.63	0.72	0.19	0.44	NR		NR					
				82.7	23.8	82.7	23.8	2.63	0.75	0.46	0.6	NR		NR					
Koseoglu et al	2013			69.6	29.31	69.6	29.31	1.62	0.52	0.42	0.63	NR		3.63	0.8	1.9	1.62	NR	
				38.3	32.64	38.3	32.64	1.72	0.71	0.78	0.71	NR		3.86	0.63	3.09	0.83	NR	
Roman et al	2013			82.25	22.2	82.25	22.2	3.74	1.45	0.83	1.19	NR		NR					
				89.75	17.33	89.75	17.33	3.32	1.36	0.41	0.7	NR		NR					
Kumar&Murthy et al	2013			77	18.42	77	18.42	2.5	0.48	0.54	0.5	NR		NR					
				83	14.5	83	14.5	2.75	0.58	0.54	0.45	NR		NR					
Cordaro et al	2012			74.8	16	74.8	16	3.12	1.11	0.81	0.56	2.31	0.87	NR					
				71	22	71	22	2.93	0.83	0.9	0.81	2.03	0.69	NR					
Cardaropoli et al	2012			94.32	11.68	94.32	11.68	3.09	0.63	0.23	0.47	-2.86	0.39	NR					
				96.97	6.74	96.97	6.74	3.05	0.65	0.09	0.2	-2.95	0.69	NR					
Alkan and Parlar et al	2011			92	14	92	14	3.5	1	0.33	0.65	3.17	0.72	3.08	0.79	0.08	0.29	3	0.85
				89	14	89	14	3.58	0.79	0.42	0.51	3.17	0.83	2.83	0.83	0.42	0.51	2.33	1.07
Jankovic et al	2010			70.5	11.76	NR		3.9	1	1.15	0.65	NR		NR					
				72.1	9.55	NR		4.1	1.05	1.05	0.45	NR		NR					
Pizzo et al	2005			90.67	16.99	90.67	16.99	4.07	0.59	0.4	0.74	NR		4.2	0.94	1.13	2	NR	
				86.67	18.29	86.67	18.29	4.13	0.74	0.6	0.83	NR		4.27	0.7	1.6	2.1	NR	
Spahr et al	2005	NR						3.597	0.834			2.767	0.829	4.337	0.775			2.887	1.612
		NR						3.813	0.841			2.39	0.893	4.423	0.815			1.957	1.729
Mcguire et al	2003			95.1		95.1		4.25	0.444	0.18		NR		6.1	1.68	NR		NR	
				93.8		93.8		4.25	0.716	0.24		NR		6.4	1.57	NR		NR	
Hagewald et al	2002			80		80		3.7	1	0.8	1	2.8	0.8	4.4	0.8			2.4	2
				79		79		3.9	1.1	1	1.2	2.9	0.9	4.4	0.9			2.2	2.2
Zuccheli et al	1998			85.7	13.8	85.7	13.8	5.8	0.7	NR		NR		NR					
				80.5	14.9	80.5	14.9	5.7	0.9	NR		NR		NR					
				93.5	8.6	93.5	8.6	5.6	0.8	NR		NR		NR					
